# Supplementary material for: An estimation of the absolute number of axons indicates that human cortical areas are sparsely connected
Source: PLoS Biol. 2022 Mar 14;20(3):e3001575. doi: 10.1371/journal.pbio.3001575 (PMC8947121; doi:10.1371/journal.pbio.3001575)
Supplement: S1 Appendix — (PDF) [file pbio.3001575.s001.pdf]

S1 Appendix: Extended Methods and Results

Diffusion MRI

The diffusion-weighted MRI (dMRI) connectivity data used to estimate inter-parcel axon totals was described in our prior report [1] whose data are available in full at https://doi.org/10.5281/zenodo.4060485. Preprocessed imaging data for 1,065 healthy young adults were retrieved from the Human Connectome Project WU-Minn consortium 1200 release [2] (https://db.humanconnectome.org). We segmented the cortex into 180 parcels per hemisphere following the HCP-MMP1.0 atlas [3] and performed probabilistic tractography using the probtrackX tool [4] from FSL [5] using tractography parameters detailed in our prior report [1]. This analysis yielded a 360 x 360 dMRI connectivity matrix for each participant which provided the example data for applying the method presented here. For probtrackX-derived connectivity, the raw unit of dMRI connectivity strength is the number of streamlines connecting seed and target regions. The absolute magnitude of this metric is arbitrary as it scales with the number of samples drawn from the probability distribution of diffusion parameters in each voxel, in our analysis 5,000.

The individual pairwise streamline counts and fiber tract length matrices for the replication analysis were described in [6] whose data are available at https://doi.org/10.5281/zenodo.4733297. These data consist of a 972 participant subset of the 1,065 WU-Minn HCP participants included in our primary analysis. Arnatkeviciute and colleagues similarly segmented the cortex following the HCP-MMP1.0 atlas [3]. In contrast to our primary dataset, tractography was performed with MRtrix3 [7] using second-order integration over fiber orientation distributions (iFOD2) [8], see [6] for details. When confined to the same participant subset and non-null connections, the pairwise tract lengths and log-transformed streamline counts are highly correlated (r^2 = 0.78 and r^2 = 0.69, respectively) between Arnatkeviciute et al. [6] and our study [1]. The rate of exponential decay in the number of streamlines vs fiber tract distance is also similar, λ = 26.1 mm in their study vs 23.4 mm in ours, though less connectivity variance is explained by distance in their results (r^2 = 0.12 vs. r^2 = 0.52). Results for these tractography data are shown in S1 Fig.

Cross-sectional area of the corpus callosum, white matter volume, and parcel areas

The HCP WU-Minn consortium 1200 release [2] includes the standard outputs from the FreeSurfer `recon-all` pipeline [9]. With default parameters, this pipeline labels the 1 mm^3 voxels of the `aseg.mgz` volume which contain the corpus callosum with a fixed 5mm lateral extent. Dividing the number of callosum-label voxels by 5 yields an estimate of its cross-sectional area, in mm^2. The total white matter volume is reported in the `aseg.stats` output of `mri_segstats`. Parcel areas and adjacency were determined [10] on triangular mesh of each participant's reconstructed white matter surface, or white matter – gray matter interface (`?h.white`). These data were used for both the primary and replication analyses.

Cross-sectional fiber packing density in corpus callosum and other hemispheric white matter

Our estimate of callosal fiber density was based on the electron microscopy (EM) study by Aboitiz and colleagues [11] who reported a fiber density of 38 per 100 μm^2 or 3.7×10^5 axons/mm^2 after accounting for tissue shrinkage to 96% volume after Epon fixation [12]. This is consistent with their finding of 2.65×10^5 axons/mm^2 as measured with light-microscopy in twenty brains as well, assuming shrinkage to 43% volume for paraffin fixation [12] and an estimated ~20% of fibers not detectable with light microscopy [11], as well as the 2.83×10^5 non-exhaustively counted axons per mm^2 reported in a more recent EM study of callosal axon diameter in two brains [13]. This value was derived by dividing the reported mean count of axons per callosal region by the callosal area surveyed, and correcting for EM preparation tissue shrinkage. An additional light microscopy study of callosal axon density in eleven control cortices reports an average of 1.12×10^5 myelinated axons per mm^2 [14], assuming shrinkage to shrinkage to 43% volume. This somewhat lower figure may be explained by the inclusion of four children's cortices in their cohort, or by mis-assumed shrinkage, but all reports are within half an order-of-magnitude of each other.

Distance matched intra- vs. inter-hemispheric dMRI connectivity

One assumption of the method described in this report is that the dMRI tractography methodology has approximately similar sensitivity to callosal and ipsilateral connections. This is difficult to determine without ground truth connectivity, as intra- and inter-hemispheric connections may have true anatomical differences.

Nevertheless, we examined distance-matched intra- vs inter-hemispheric connections (Fig 1*C*) and found no statistical evidence that they are different. Pairwise connections were averaged in 15 distance bins, log-transformed and linear fits obtained for intra- and inter-hemispheric connections. For the overlapping distance domain, the intra-hemispheric slope is -0.30, 95% confidence interval = [-0.33 -0.26] with an intercept of 6.18 [5.91 6.45] and the inter-hemispheric slope is -0.26 [-0.29 -0.24] and intercept of 5.93 [5.72 6.14]; the 95% confidence intervals of both parameters are overlapping. In addition, a paired r-test of correlation differences [15] failed to reject the null hypothesis, $p = 0.58$. The parity between distance-matched intra- and inter-hemispheric connections also holds when the linear fit is obtained for over the entire distance domain, with an intra-hemispheric slope and intercept -.31 [-0.33 -0.28] and 6.24 [6.01 6.46], respectively; -.28 [-0.30 -0.25] and 6.00 [5.78 6.23] for inter-hemispheric connections. In the replication analysis with alternative tractography data [6] (S1 Fig C) the paired r-test likewise failed to reject the null hypothesis, $p = 0.34$. The intra-hemispheric slope and intercept are -.37 [-0.37 -0.78] and 4.19 [3.25 5.13], respectively; -.25 [-0.30 -0.20] and 3.30 [2.82 3.79] for inter-hemispheric connections.

Estimating axon counts and axon volume from dMRI

From the histological estimate of callosal fiber density, MRI-derived measures of callosal cross-sectional area and white matter connectivity, the conversion factor between dMRI connectivity and physical axons can be estimated as follows:

Eq. 1.1 $N_{callosum} = A_{callosum} * D_{callosum}$

Eq. 1.2 $C_{axon} = \frac{Ncallosum}{M_{dMRI}}$

Where $A_{callosum}$ is the cross-sectional area (mm^2), $D_{callosum}$ is the assumed fiber density ($3.7x10^5$ axons/mm^2) of the corpus callosum, $N_{callosum}$ is the estimated total number of callosal fibers, M_{dMRI} is the magnitude of dMRI callosal connectivity, in our case, the number of tractography streamlines, and C_{axon} is the conversion factor from dMRI to physical fibers (axons/streamline).

In addition to connectivity strength, or number of streamlines, the dMRI tractography analysis also yields a mean streamline length for every parcel-pair connection. After converting dMRI connectivity strength to physical axon count as described above, the total volume of the estimated axons can be calculated as follows:

Eq. 2.1 $WM_{volume} = \sum_{i=1}^{N_{pair}} l_i * n_i * C_{axon} * \frac{1}{D_{callosum}}$

Eq. 2.2 $WM_{volume} = \sum_{i=1}^{N_{pair}} l_i * n_i * \frac{A_{callosum}}{M_{dMRI}}$

Where WM_{volume} is the estimated axonal white matter volume (mm^3) and N_{pair} is the total number of parcel pairs. l_i and n_i are the length (mm) and number of dMRI streamlines in the ith parcel pair. Eq. 2.1 can be simplified into Eq. 2.2 by substituting Eqs. 1.1 and 1.2 into 2.1.

Parcel grouping into functional networks

The parcel ordering and the ten functional networks shown in Fig 1*A* were defined in our previous report [1] and were modified from twelve networks identified by Ji and colleagues in a resting-state fMRI study on the HCP cohort [16] by merging the primary and secondary visual networks as well as the ventral and posterior multimodal networks.

Effects of sex and age on inter-hemispheric connectivity

In their histological study of 20 cortices , Aboitiz and colleagues [11] found no significant effects of sex or age on the total number of fibers, fiber density, and cross-sectional area of the corpus callosum. As we assume a constant fiber density, our estimate of the total number of inter-hemispheric fibers is a linear function of the callosal cross-sectional area. As the ages of the HCP 1200 cohort are grouped into four broad ranges in the open access data, we investigated the effects of sex and age on the estimated number inter-hemispheric axons with 2x4 fixed effects ANOVA, treating the age group factor as categorical. Mean and individual values are shown in S2 Fig. Note that while the number of pairwise axons is approximately log-normally distributed across areal pairs, it is approximately normally distributed across individuals.

We found that, on average, males have $2.61x10^8$ inter-hemispheric axons, 95% confidence interval = [$2.58x10^8$ $2.64x10^8$] and females have $2.50x10^8$ [$2.47x10^8$ $2.52x10^8$] axons. Participants ages 22-25, 26-30, 31-35, and 36+

year old have 2.50×10^8 [2.46×10^8 2.55×10^8], 2.58×10^8 [2.55×10^8 2.61×10^8], 2.55×10^8 [2.51×10^8 2.59×10^8], and 2.35×10^8 [2.15×10^8 2.54×10^8] axons, respectively. Intervals show bootstrapped 95% confidence. We find no effect of sex on the number of inter-hemispheric axons, $F_{1,1064} = 3.471$, $p = 0.0627$. Removing the one female outlier does not bring the mean difference into statistical significance, $F_{1,1063} = 3.653$, $p = 0.0562$. The interaction of sex and age on the number of inter-hemispheric axons is likewise not significant $F_{3,1064} = 2.282$, $p = 0.0776$. Age does significantly affect the number of inter-hemispheric neurons, $F_{3,1064} = 5.100$, $p = 0.0017$. Post-hoc pairwise tests show that only the difference between the 22-25 and 26-30 year old age groups is significant, $F_{1,1064} = 7.646$, $p = 0.0058$. These results are broadly consistent with those of Aboitiz et al. [11]. A more in-depth examination of the effects of age on corpus callosum requires a cohort with which includes more older participants as well as more precise age values.

Thalamo-cortical fiber volume estimation

The scope of our previous dMRI tractography study [1] was confined to cortico-cortical (including cortico-hippocampal) connectivity. In order to estimate the total volume of thalamo-cortical axons, we first calculated the number of excitatory thalamic neurons by summing the total number of neurons in the thalamus [17], excluding the reticular nucleus, zona incerta, limitans/suprageniculate, and subthalamus, and including from the remainder the ~62% proportion of thalamic neurons that are excitatory [18]. The number of such neurons is 22.6×10^6. We assumed that each such thalamic neuron projects a single axon to the cortex, and we allocated these axons to cortical parcels in proportion to their area. The inverse callosal packing density was used for effective cross-sectional area, as with cortico-cortical connections. To estimate the length of thalamo-cortical fiber tracts, for each parcel i, we first identified the thalamic voxel nearest to parcel's centroid. We then used the inter-parcel fiber tract length [1] from the centroid of parcel i to the identified parathalamic parcel centroid. To this figure we added the Euclidean distance from the parathalamic centroid to the nearest voxel of the thalamus, ensuring that estimated thalamo-cortical fiber tract length is always non-zero. As with cortico-cortical connections, the total white matter volume of each connection was estimated by taking the product of the number of axons, the effective cross-sectional area, and the estimated fiber tract length (Eq. 2).

Estimation of proportion of actual inter-areal axons to the number that would be needed for complete inter-connectivity

We estimated that the total number of inter-areal axons is ~2.43×10^9. Assuming $n = 16.34 \times 10^9$ total cortical neurons (including interneurons) [19] , the total number of connections needed for complete whole-cortex connectivity is $n(n-1) = 2.67 \times 10^{20}$. We estimated the neural density of the cortex as ~92,300 neurons/mm^2 by dividing 16.34×10^9 by 1.77×10^5 mm^2 mean white—gray surface area of the HCP cohort used. The total number of connections needed for complete inter-connectivity within each area is approximately 1.18×10^{18}, given by

Eq. 3.1 $$N_{completeIntra-areal} = \sum_{i=1}^{N_{parcel}} \left(A_{parcel} * D_{neuron}\right)^2$$

Where A_{parcel} is the area of each parcel and D_{neuron} the neural density. Subtracting the connections needed for within-area inter-connectivity from whole cortex interconnectivity yields 2.66×10^{20}, which is 1.10×10^{11} times greater than the number of inter-areal contacts we calculate with our method.

Number of neurons in non-cortical regions communicating with the cortex

The number of neurons in different human brain regions has been reviewed by Blinkov and Glexer [20] and von Bartheld et al. [21]. The number of neurons in the amygdala have been estimated as ~13×10^6 [22], striatum as ~55×10^6 [23], and thalamus as ~23×10^6 (thalamocortical, see above). These are the only recipients or targets of cortical connections with significant numbers of neurons. Other locations, such those which are the origins of serotonin [24], norepinephrine [25], dopamine [26], or acetylcholine [27] afferents to the cortex all have ~50,000 axons each. Summed together, the number of cells in all of the above structures comes to ~0.5% of the number of cortical cells. Direct projections outside of the brain are exceedingly rare, with the number of Betz cells in the primary motor cortex [28] or medullary pyramid [29] both estimated as ~100,000. It is difficult to escape the conclusion that, at least in humans, the cortex communicates mainly with itself.

References

1. Rosen BQ, Halgren E. A Whole-Cortex Probabilistic Diffusion Tractography Connectome. eNeuro. 2021;8: ENEURO.0416-20.2020. doi:10.1523/ENEURO.0416-20.2020
2. Van Essen DC, Smith SM, Barch DM, Behrens TEJ, Yacoub E, Ugurbil K. The WU-Minn Human Connectome Project: An overview. Neuroimage. 2013;80: 62–79. doi:10.1016/j.neuroimage.2013.05.041
3. Glasser MF, Coalson TS, Robinson EC, Hacker CD, Harwell J, Yacoub E, et al. A multi-modal parcellation of human cerebral cortex. Nature. 2016;536: 171–8. doi:10.1038/nature18933
4. Behrens TEJ, Berg HJ, Jbabdi S, Rushworth MFS, Woolrich MW. Probabilistic diffusion tractography with multiple fibre orientations: What can we gain? Neuroimage. 2007;34: 144–155. doi:10.1016/j.neuroimage.2006.09.018
5. Jenkinson M, Beckmann CF, Behrens TEJ, Woolrich MW, Smith SM. FSL. Neuroimage. 2012;62: 782–790. doi:10.1016/j.neuroimage.2011.09.015
6. Arnatkeviciute A, Fulcher BD, Oldham S, Tiego J, Paquola C, Gerring Z, et al. Genetic influences on hub connectivity of the human connectome. Nat Commun. 2021;12: 4237. doi:10.1038/s41467-021-24306-2
7. Tournier JD, Smith R, Raffelt D, Tabbara R, Dhollander T, Pietsch M, et al. MRtrix3: A fast, flexible and open software framework for medical image processing and visualisation. Neuroimage. 2019;202: 116137. doi:10.1016/j.neuroimage.2019.116137
8. Tournier JD, Calamante F, Connelly A. Improved probabilistic streamlines tractography by 2nd order integration over fibre orientation distributions. Proceedings of the international society for magnetic resonance in medicine. New Jersey, USA: John Wiley & Sons, Inc.; 2010.
9. Fischl B. FreeSurfer. Neuroimage. 2012;62: 774–781. doi:10.1016/j.neuroimage.2012.01.021
10. Meyer M, Desbrun M, Schröder P, Barr AH. Discrete Differential-Geometry Operators for Triangulated 2-Manifolds. 2003. pp. 35–57. doi:10.1007/978-3-662-05105-4_2
11. Aboitiz F, Scheibel AB, Fisher RS, Zaidel E. Fiber composition of the human corpus callosum. Brain Res. 1992;598: 143–153. doi:10.1016/0006-8993(92)90178-C
12. Schüz A, Palm G. Density of neurons and synapses in the cerebral cortex of the mouse. J Comp Neurol. 1989;286: 442–455. doi:10.1002/cne.902860404
13. Liewald D, Miller R, Logothetis N, Wagner HJ, Schüz A. Distribution of axon diameters in cortical white matter: an electron-microscopic study on three human brains and a macaque. Biol Cybern. 2014;108: 541–557. doi:10.1007/s00422-014-0626-2
14. Wegiel J, Flory M, Kaczmarski W, Brown WT, Chadman K, Wisniewski T, et al. Partial Agenesis and Hypoplasia of the Corpus Callosum in Idiopathic Autism. J Neuropathol Exp Neurol. 2017;76: 225–237. doi:10.1093/jnen/nlx003
15. Williams EJ. The comparison of regression variables. J R Stat Soc Ser B. 1959;21: 396–399.
16. Ji JL, Spronk M, Kulkarni K, Repovš G, Anticevic A, Cole MW. Mapping the human brain's cortical-subcortical functional network organization. Neuroimage. 2019;185: 35–57. doi:10.1016/j.neuroimage.2018.10.006
17. Xuereb JH, Perry RH, Candy JM, Perry EK, Bonham JR. Nerve cell Loss in the thalamus in Alzheimer's disease and Parkinson's disease. Brain. 1991;114: 1363–1379. doi:10.1093/brain/114.3.1363
18. Arcelli P, Frassoni C, Regondi MC, De Biasi S, Spreafico R. GABAergic neurons in mammalian thalamus: a marker of thalamic complexity? Brain Res Bull. 1997;42: 27–37. doi:10.1016/S0361-9230(96)00107-4
19. Azevedo FAC, Carvalho LRB, Grinberg LT, Farfel JM, Ferretti REL, Leite REP, et al. Equal numbers of neuronal and nonneuronal cells make the human brain an isometrically scaled-up primate brain. J Comp Neurol. 2009;513: 532–541. doi:10.1002/cne.21974
20. Blinkov SM, Glezer II. The human brain in figures and tables: a quantitative handbook. Basic Books; 1968.
21. von Bartheld CS, Bahney J, Herculano-Houzel S. The search for true numbers of neurons and glial cells in the human brain: A review of 150 years of cell counting. J Comp Neurol. 2016;524: 3865–3895. doi:10.1002/cne.24040
22. Avino TA, Barger N, Vargas M V, Carlson EL, Amaral DG, Bauman MD, et al. Neuron numbers increase in the human amygdala from birth to adulthood, but not in autism. Proc Natl Acad Sci. 2018;115: 3710–3715.
23. Beckmann H, Lauer M. The human striatum in schizophrenia. II. Increased number of striatal neurons in schizophrenics. Psychiatry Res Neuroimaging. 1997;68: 99–109. doi:10.1016/s0925-4927(96)02947-2
24. Baker KG, Halliday GM, Hornung J-P, Geffen LB, Cotton RGH. Distribution, morphology and number of monoamine-synthesizing and substance P-containing neurons in the human dorsal raphe nucleus. Neuroscience. 1991;42: 757–775. doi:10.1016/0306-4522(91)90043-n
25. Mouton PR, Pakkenberg B, Gundersen HJG, Price DL. Absolute number and size of pigmented locus coeruleus neurons in young and aged individuals. J Chem Neuroanat. 1994;7: 185–190. doi:10.1016/0891-0618(94)90028-0
26. Eriksen N, Stark AK, Pakkenberg B. Age and Parkinson's disease-related neuronal death in the substantia nigra pars compacta. Birth, life death dopaminergic neurons Subst Nigra. 2009; 203–213. doi:10.1007/978-3-211-92660-4_16
27. Mufson EJ, Ginsberg SD, Ikonomovic MD, DeKosky ST. Human cholinergic basal forebrain: chemoanatomy and neurologic dysfunction. J Chem Neuroanat. 2003;26: 233–242. doi:10.1016/s0891-0618(03)00068-1

28. Rivara C, Sherwood CC, Bouras C, Hof PR. Stereologic characterization and spatial distribution patterns of Betz cells in the human primary motor cortex. Anat Rec Part A Discov Mol Cell Evol Biol An Off Publ Am Assoc Anat. 2003;270: 137–151. doi:10.1002/ar.a.10015
29. Wada A, Goto J, Goto N, Kawamura N, Matsumoto K. Are There One Million Nerve Fibres in the Human Medullary Pyramid? Okajimas Folia Anat Jpn. 2001;77: 221–224. doi:10.2535/ofaj1936.77.6_221
