# Supplementary material for: An estimation of the absolute number of axons indicates that human cortical areas are sparsely connected
Source: PLoS Biol. 2022 Mar 14;20(3):e3001575. doi: 10.1371/journal.pbio.3001575 (PMC8947121; doi:10.1371/journal.pbio.3001575)
Supplement: S1 Fig — Results obtained by repeating the analysis using an alternative tractography dataset [15]. (A) Connectivity matrix of intrahemispheric axon counts, averaged across the 2 hemispheres. Parcels are ordered into 10 functional networks [11]. (B) Histograms showing the distribution of inter- and intrahemispheric pairwise axon counts. Physically adjacent and nonadjacent parcel pair intrahemispheric histograms are stacked. Median connectivity, shown in gray, is approximately 2,500 axons between cortical areas in the same hemisphere and approximately 300 interhemispherically. Parcel pairs with zero axons connecting them are represented by the bars left of the y-axis. (C) dMRI connectivity as a function fiber tract distance. Pairwise values averaged within 15 fiber length bins. Shading shows bootstrapped 95% confidence intervals. (D–F) Axons counts and densities averaged across the 2 hemispheres and visualized on the left fsaverage template cortex [12]. (D) Total axons connecting each parcel to all others. (E) Axons connecting each parcel to all others, normalized by the reference parcel’s area. (F) Axons connecting the hippocampus to the rest of the cortex, normalized by the area of the cortical parcel, shown in log scale. dMRI, diffusion MRI. (PDF) [file pbio.3001575.s002.pdf]

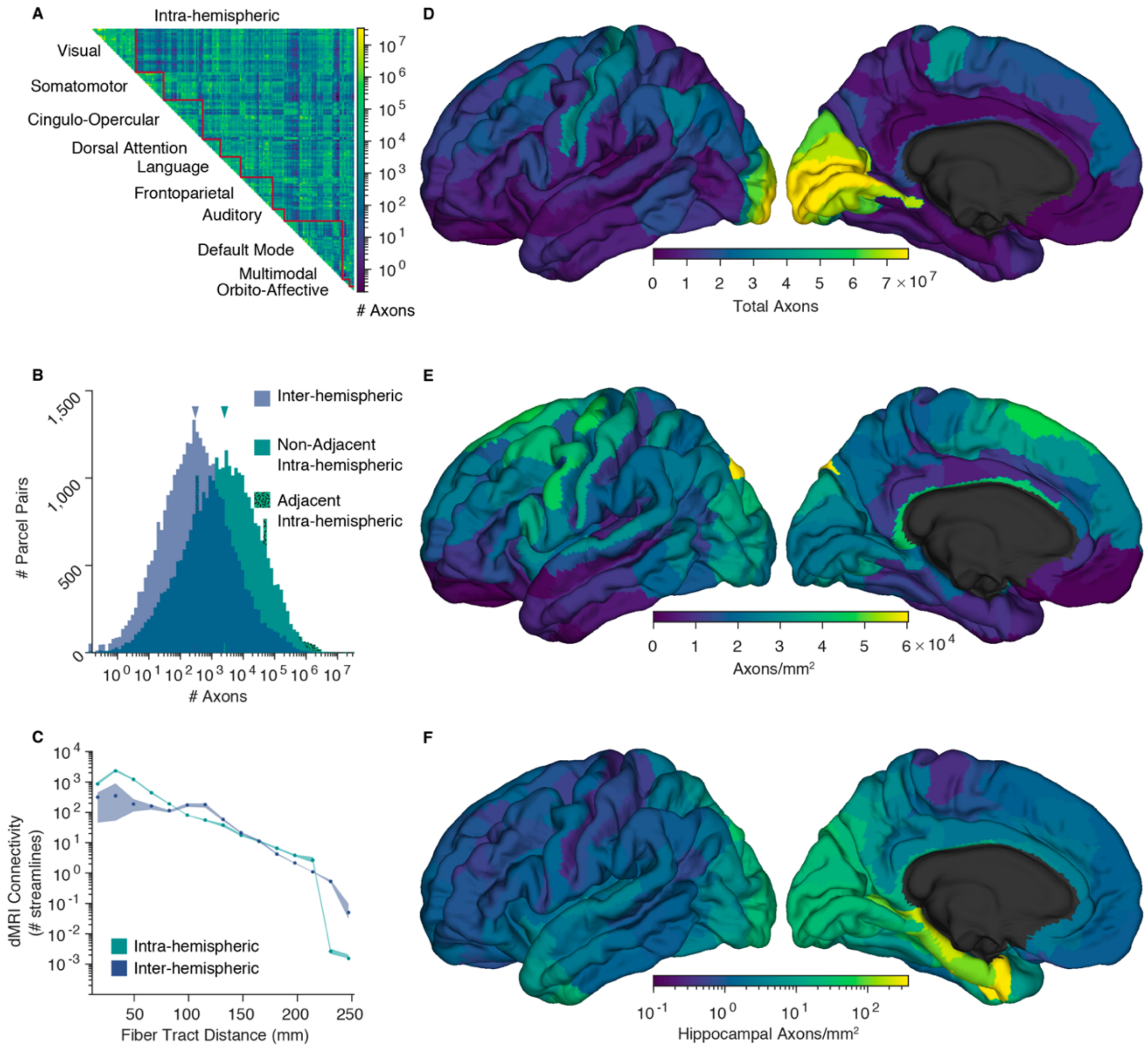

S1 Fig: Replication results. Results obtained by repeating the analysis using an alternative tractography dataset [1] (A) Connectivity matrix of intra-hemispheric axon counts, averaged across the two hemispheres. Parcels are ordered into 10 functional networks [2]. (B) Histograms showing the distribution of inter- and intra-hemispheric pairwise axon counts. Physically adjacent and non-adjacent parcel pair intra-hemispheric histograms are stacked. Median connectivity, shown in gray, is ~2,500 axons between cortical areas in the same hemisphere, and ~300 inter-hemispherically. Parcel pairs with zero axons connecting them are represented by the bars left of the y-axis. (C) dMRI connectivity as a function fiber tract distance. Pairwise values averaged within 15 fiber-length bins. Shading shows bootstrapped 95% confidence intervals. (D-F) axons counts and densities averaged across the two hemispheres and visualized on the left fsaverage template cortex [3]. (D) total axons connecting each parcel to all others. (E) axons connecting each parcel to all others, normalized by the reference parcel's area. (F) axons connecting the hippocampus to the rest of the cortex, normalized by the area of the cortical parcel, shown in log scale.

1. Arnatkeviciute A, Fulcher BD, Oldham S, Tiego J, Paquola C, Gerring Z, et al. Genetic influences on hub connectivity of the human connectome. Nat Commun. 2021;12: 4237. doi:10.1038/s41467-021-24306-2
2. Rosen BQ, Halgren E. A Whole-Cortex Probabilistic Diffusion Tractography Connectome. eNeuro. 2021;8: ENEURO.0416-20.2020. doi:10.1523/ENEURO.0416-20.2020
3. Fischl B. FreeSurfer. Neuroimage. 2012;62: 774–781. doi:10.1016/j.neuroimage.2012.01.02
