# Supplementary material for: An estimation of the absolute number of axons indicates that human cortical areas are sparsely connected
Source: PLoS Biol. 2022 Mar 14;20(3):e3001575. doi: 10.1371/journal.pbio.3001575 (PMC8947121; doi:10.1371/journal.pbio.3001575)
Supplement: S2 Fig — Each individual’s total number of estimated interhemispheric axons is shown with a marker. The black horizonal bars show the group means and vertical bars the bootstrapped 95% confidence intervals these means. Shading shows a kernel density estimate of the group distributions. Note that while the number of pairwise axons is approximately log normally distributed across areal pairs, it is approximately normally distributed across individuals. The only significant group difference is between the 22 to 15 and 25 to 30 age groups, F1,1064 = 7.646, p = 0.0058. Interactions between sex and age effect were not significant. As we assume a constant fiber density, our estimate the total number of interhemispheric fibers is a linear multiple of the callosal cross-sectional area. (PDF) [file pbio.3001575.s003.pdf]

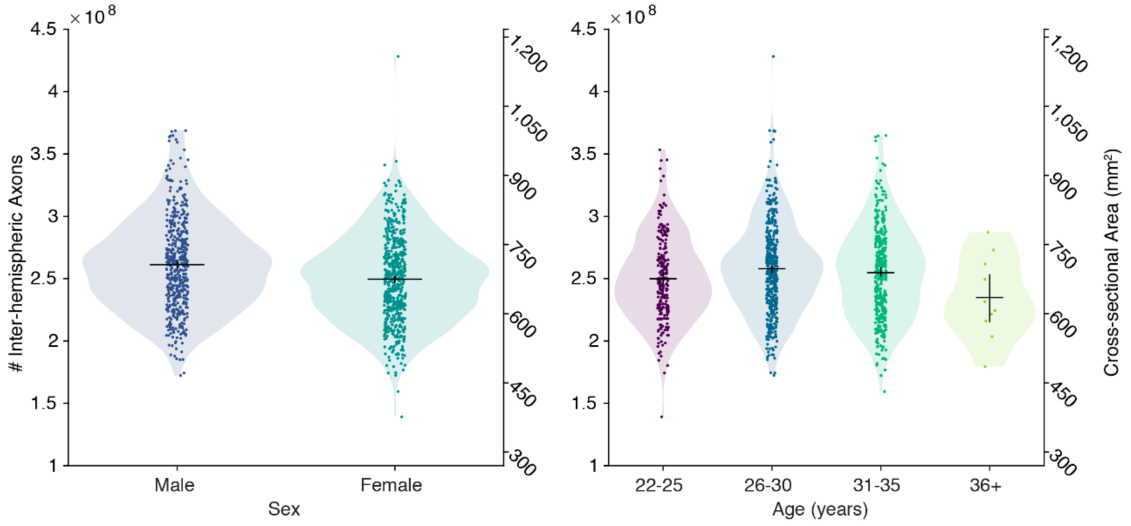

S2 Fig: Effects of sex and age on the inter-hemispheric connectivity. Each individual's total number of estimated inter-hemispheric axons is shown with a marker. The black horizonal bars show the group means and vertical bars the bootstrapped 95% confidence intervals these means. Shading shows a kernel density estimate of the group distributions. Note that while the number of pairwise axons is approximately log-normally distributed across areal pairs, it is approximately normally distributed across individuals. The only significant group difference is between the 22-15 and 25-30 age groups, $F_{1,1064} = 7.646$, $p = 0.0058$. Interactions between sex and age effect were not significant. As we assume a constant fiber density, our estimate the total number of inter-hemispheric fibers is a linear multiple of the callosal cross-sectional area.
