# Supplementary material for: An estimation of the absolute number of axons indicates that human cortical areas are sparsely connected
Source: PLoS Biol. 2022 Mar 14;20(3):e3001575. doi: 10.1371/journal.pbio.3001575 (PMC8947121; doi:10.1371/journal.pbio.3001575)
Supplement: S3 Fig — In order to estimate the fraction of tract axons that travel the entire length of the tract, its termination fields, centered on Broca’s and Wernicke’s areas, were manually defined in terms of HCP-MMP1.0 parcels [16] according to consensus definitions [22]. (A) Conservative definitions where the anterior termination field, in teal, is composed of parcels 44, 45, 6r, IFSa, IFSp, and FOP4 and the posterior termination field, in yellow, is composed of parcels PSL, RI, STV, and PFcm. For this definition, trans-terminal axons account for 0.6% and 0.8% of tract axons in the left and right hemispheres. (B) Liberal definitions in which parcels 47l and p47r were added anteriorly and parcels PF, PFm, and PGi were added posteriorly, resulting in trans-terminal axons accounting for 1.9% and 2.9% of tract axons in the left and right hemispheres. Gray lines indicate the approximate locations at which the tract diameter, used to estimate the total number of tract axons were ascertained [21]. Areas are rendered on the fsaverage template cortex [12]. AF/SLF, arcuate/superior lateral fasciculus; HCP, Human Connectome Project. (PDF) [file pbio.3001575.s004.pdf]

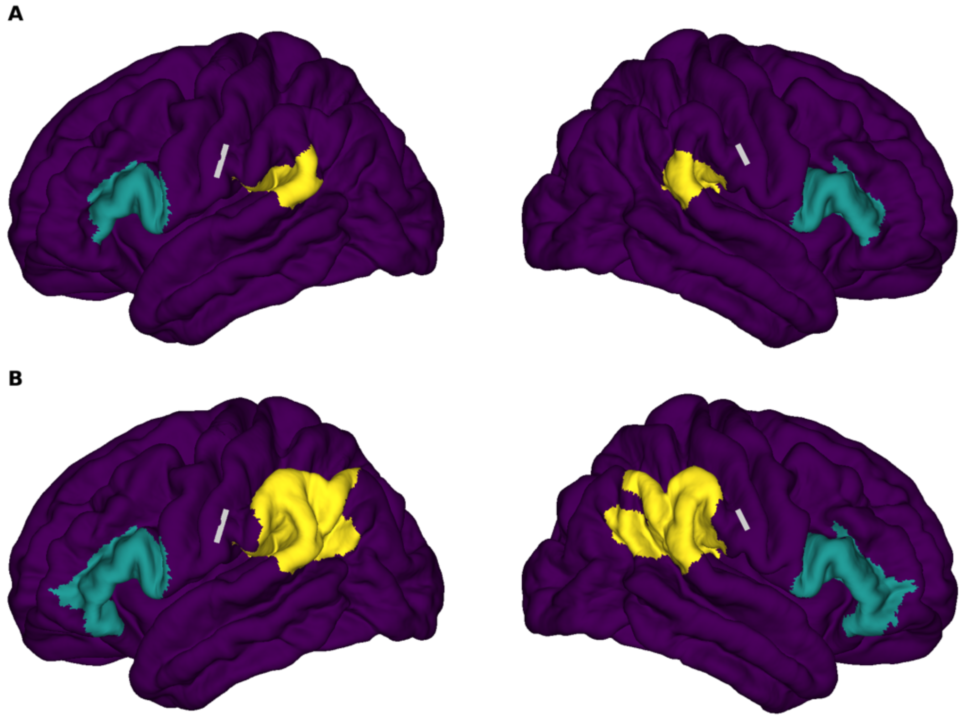

S3 Fig: Arcuate / superior lateral fasciculus termination fields. In order to estimate the fraction of tract axons that travel the entire length of the tract, it's termination fields, centered on Broca's and Wernicke's areas, were manually defined in terms of HCP-MMP1.0 parcels [1] according to consensus definitions [2]. (A) Conservative definitions where the anterior termination field, in teal, is composed of parcels 44, 45, 6r, IFSa, IFSp, and FOP4 and the posterior termination field, in yellow, is composed of parcels PSL, RI, STV, and PFcm. For this definition trans-terminal axons account for 0.6% and 0.8% of tract axons in the left and right hemispheres. (B) Liberal definitions in which parcels 47l and p47r were added anteriorly and parcels PF, PFm, and PGi were added posteriorly, resulting in trans-terminal axons accounting for 1.9% and 2.9% of tract axons in the left and right hemispheres. Gray lines indicate the approximate locations at which the tract diameter, used to estimate the total number of tract axons were ascertained [3]. Areas are rendered on the fsaverage template cortex [4].

1. Glasser MF, Coalson TS, Robinson EC, Hacker CD, Harwell J, Yacoub E, et al. A multi-modal parcellation of human cerebral cortex. Nature. 2016;536: 171–8. doi:10.1038/nature18933
2. Tremblay P, Dick AS. Broca and Wernicke are dead, or moving past the classic model of language neurobiology. Brain Lang. 2016;162: 60–71. doi:https://doi.org/10.1016/j.bandl.2016.08.004
3. Yeh F-C. Shape analysis of the human association pathways. Neuroimage. 2020;223: 117329. doi:https://doi.org/10.1016/j.neuroimage.2020.117329
4. Fischl B. FreeSurfer. Neuroimage. 2012;62: 774–781. doi:10.1016/j.neuroimage.2012.01.021
