# Supplementary material for: An estimation of the absolute number of axons indicates that human cortical areas are sparsely connected
Source: PLoS Biol. 2022 Mar 14;20(3):e3001575. doi: 10.1371/journal.pbio.3001575 (PMC8947121; doi:10.1371/journal.pbio.3001575)
Supplement: S4 Fig — In order to explore the possibility that dMRI tractography is less sensitive to shorter connections the streamline-to-axon ratio was increased linearly with inverse fiber length using a range of slope parameters. Packing density was assumed to be reciprocally decreased in order to fix a constant total cerebral WM volume. (A) Distributions of intra- and interhemispheric interareal axons counts. Gray histograms show the effect of uniformly halfling the ratio and doubling the assumed packing density. (B) Intrahemispheric and (C) interhemispheric adjustments to the # of pairwise axons as a function of inverse fiber tract length and the resultant increase in median axon count as a function of the adjustments’ slope parameter. At slope = 0, values are unadjusted from the primary analysis. Large adjustments only increase median counts modestly in the context of the log-normal distribution. For example, for intrahemispheric connections (B), at a slope of 0.004, the number of the shortest axons is about doubled (i.e., corresponding to doubling the axon density and halving the sensitivity of dMRI to axons), but the median number of interparcel axons only increases by approximately 36%. dMRI, diffusion MRI; WM, white matter. (PDF) [file pbio.3001575.s005.pdf]

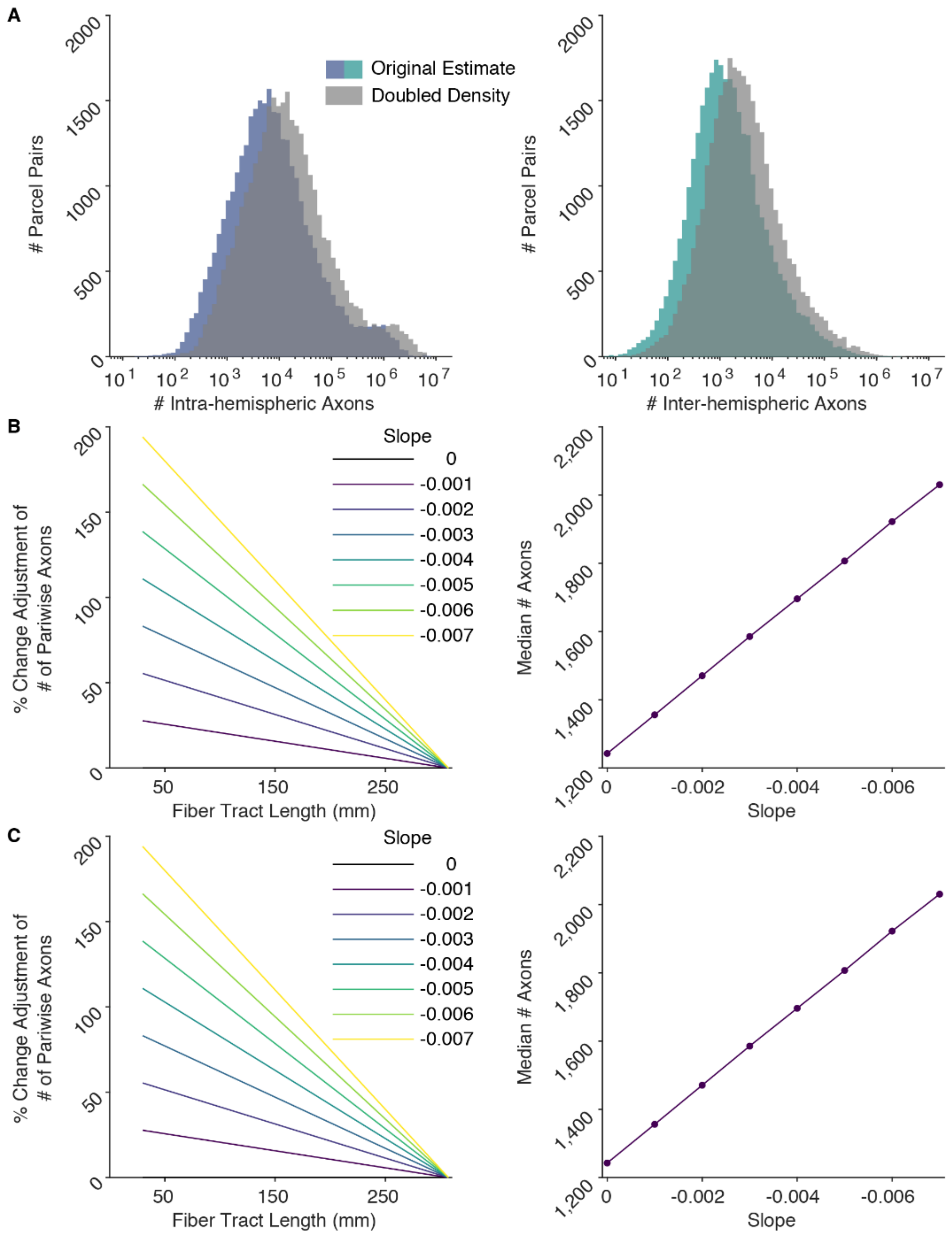

S4 Fig: Simulated effect of short axon underestimation on pairwise axon counts. In order to explore the possibility that dMRI tractography is less sensitive to shorter connections the streamline-to-axon ratio was increased linearly with inverse fiber length using a range of slope parameters. Packing density was assumed to be reciprocally decreased in order to fix a constant total cerebral white matter volume. (A) Distributions of Intra- and inter-hemispheric inter-areal axons counts. Gray histograms show the effect of uniformly halfling the ratio and doubling the assumed packing density. (B) intra-hemispheric and (C) inter-hemispheric adjustments to the # of pairwise axons as a function of inverse fiber tract length and the resultant increase in median axon count as a function of the adjustments' slope parameter. At slope = 0, values are unadjusted from the primary analysis. Large adjustments only increase median counts modestly in the context of the log-normal distribution. For example, for intra-hemispheric connections (B), at a slope of 0.004, the number of the shortest axons is about doubled (i.e., corresponding to doubling the axon density and halving the sensitivity of dMRI to axons), but the median number of inter-parcel axons only increases by ~36%.
