# Supplementary material for: An estimation of the absolute number of axons indicates that human cortical areas are sparsely connected
Source: PLoS Biol. 2022 Mar 14;20(3):e3001575. doi: 10.1371/journal.pbio.3001575 (PMC8947121; doi:10.1371/journal.pbio.3001575)
Supplement: Attachment — Submitted filename: Review on the manuskript Rosen Halgren.pdf [file pbio.3001575.s006.pdf]

Review on the Manuscript:

Human cortical areas are sparsely connected: Combining histology with diffusion MRI to estimate the absolut number of axons, by Rosen and Halgren

Summary

This is a fascinating paper. It quantifies in an elegant way cortico-cortical connections between distant cortical areas in the human brain. The results are in support of findings which indicate a preponderance of connectivity between closely located areas (Schüz and Braiten-berg, 2002), also in other species (Scannell et al., 1995; Schüz at al., 2006). The study by Rosen and Halgren is outstanding since - in contrast to previous studies – it is able to provide an astonishingly concrete estimate for the median number of axons between distant cortical areas in the human brain.

The study uses the parcellation into 180 areas in each hemisphere by Glasser et al. (2016). This parcellation is based on a combination of neuroanatomical (mainly myelin) and functional features, by way of MRI and fMRI. The number of areas comes close to that of the myeloarchitectonic areas by the Vogt and Vogt school. The present study is based on diffusion MRI data from the database of the Human Connectome Project.

In this paper, the relative connectivity provided by dMRI (number of streamlines) is transformed into absolute numbers of axons. This transformation is based on a comparison with histological data from the literature on the density of axons in the Corpus callosum (Aboitiz et al, 1992). It leads to a conversion factor of 0.87 axons per streamline. The authors assume that the same factor can be applied to both, the Corpus callosum and to the other long range systems via the white matter. This is a reasonable assumption.

Presentation of data:

It would be good to visualize not only the median:

The HCP data contain a family structure with genetic related and unrelatedness and many other behavioral measures (Van Essen et al., 2012). The data also vary with age (22-36 years). Thus, the number of streamlines (Page 11, Line 292) between cortical areas shows inter-individual variability, affecting axon estimation. It would be essential to visualize a scatter plot (e.g. for the Corpus callosum) how the spread of the number of axons is depicted in the healthy HCP sample. Are these values in an acceptable range? As tractography relies on coarser spatial resolution, partial volume effects, and may be erroneous due to false-positive/negative estimation of streamlines.

The conversion factor

The conversion factor is the crucial point in this paper. Re-reading Aboitiz' paper and based on my own histological experience I come to the conclusion that your cobersion factor is at the lower end and is rather around 1.6. This does not invalidate the paper – a factor of 2 is negligible in this kind of statistical neuroanatomy – but it gives an idea of the possible range.

Let me explain. In Line 307 to 313 you describe your approach. In line 308 you say "electron microscopic study", but it is both light and electron microscopic. The shrinkage factor mentioned in the method's part of Aboitiz' paper is only valid for his light microscopic material, embedded in paraffin. He does not mention any shrinkage factor for his electron microscopic material (embedded in Epon), and – according to our own experience – there is hardly any shrinkage in such material. (The volume in our EM-material is about 96% of the original tissue after fixation; Schüz and Palm. 1989).

The number you mention for light microscopy of $1.57x10^5/mm^2$ is not mentioned explicitly in Aboitiz paper as far as I can see, but you probably calculated it from the data given in his table I and corrected it for areal shrinkage. Correct?

Aboitiz estimates that about 20% of fibers were not detected in the light microscope. So we end up with a range of about $1.6x10^5/mm^2$ from light microscopy and about $3.8 \times 10^5/mm^2$ from electron microscopy. The reality is probably somewhere between these values.

This is supported when looking at the total number of axons in the Corpus callosum. Aboitiz estimates 2×10^8 fibers. This is twice the number you get when using his light microscopic density of about $1.6x10^5 mm^2$ and your average areal size. (He does not give an areal size as far as I can see). This speaks in favour of a density between the LM and EM-data, and it leads to a conversion factor of 1.6 rather than 0.87.

The inverse packing density (area per axon) in line 104 would then be lower, but well within the possible range. The average axonal diameter is below 1 μm in most cortico-cortical long-range systems (Liewald et al, 2014).

Some points to be clarified

In the discussion in lines 130 and in line 165 the authors quote Liewald et al. (2014) for an alternative value for packing density in the corpus callosum of $1.23 \times 10^5 /mm^2$. I cannot find this number in the quoted paper. Did the authors somehow calculate this value from the fiber diameters given there? Or did I overlook something?

Another point: in line 176 the authors quote Azevedo et al. (2009) for a number of $11.5x10^9$ cortical pyramidal cells. I cannot find a number for cortical pyramidal cells in this paper. Did the authors derive this from the total number of cortical neurons mentioned on p.535 ($16.34x10^9$) and perhaps subtract a percentage of non-pyramidal cells?

Also, in some cases the same paper is quoted under 2 different numbers in the reference list: Liewald et al. under 12 and 14, Aboitiz et al under 5 and 11.

Finally, on line 107 the names Schüz and Braitenberg are misprinted. (And thanks to this quotation I discovered a serious printing error in our own paper: on p.381, first line, it should be $6x10^9$ not $6x10^3$)

References:

Aboitiz at al. (1992), as quoted under [5] and [11]

Glasser et al. (2016), as quoted under [7]

Liewald et al. (2014), as quoted under [12] and [14]

Scannell MP, Blakemore C, Young MP (1995) Analysis of connectivity in the cat cerebral cortex. The J. of Neuroscience 15, 1463-1483

Schüz A.**,** Chaimow D, Liewald D and Dortenmann M (2006) Quantitative Aspects of Corticocortical Connections: A Tracer Study in the Mouse. Cerebral Cortex October 2006; 16:1474--1486 , doi:10.1093/cercor/bhj085

Schüz and Braitenberg (2002), as quoted under [8]

Schüz A. and Palm G (1989) Density of neurons and synapses in the cerebral cortex of the mouse. The J. Comp. Neurol. 286: 442-455

Glasser, M.F., Coalson, T.S., Robinson, E.C., Hacker, C.D., Harwell, J., Yacoub, E., Ugurbil, K., Andersson, J., Beckmann, C.F., Jenkinson, M., Smith, S.M., Essen, D.C.V., 2016. A multi-modal parcellation of human cerebral cortex. Nature 536, 171–178. https://doi.org/10.1038/nature18933

Van Essen, D.C., Ugurbil, K., Auerbach, E., Barch, D., Behrens, T.E.J., Bucholz, R., Chang, A., Chen, L., Corbetta, M., Curtiss, S.W., Della Penna, S., Feinberg, D., Glasser, M.F., Harel, N., Heath, A.C., Larson-Prior, L., Marcus, D., Michalareas, G., Moeller, S., Oostenveld, R., Petersen, S.E., Prior, F., Schlaggar, B.L., Smith, S.M., Snyder, A.Z., Xu, J., Yacoub, E., WU-Minn HCP Consortium, 2012. The Human Connectome Project: a data acquisition perspective. NeuroImage 62, 2222–2231. https://doi.org/10.1016/j.neuroimage.2012.02.018
