# Supplementary material for: An estimation of the absolute number of axons indicates that human cortical areas are sparsely connected
Source: PLoS Biol. 2022 Mar 14;20(3):e3001575. doi: 10.1371/journal.pbio.3001575 (PMC8947121; doi:10.1371/journal.pbio.3001575)
Supplement: Attachment — Submitted filename: Numbers_PLoSBIO_respReviewers_v8_clean.pdf [file pbio.3001575.s008.pdf]

First, we thank the editor and reviewers for their careful examination of our manuscript and believe addressing these concerns has resulted in a much stronger paper.

We appreciate that the reviewers' foremost concern is the extrapolation of the (axon-count)/(dMRI-streamline count) ratio from the corpus callosum to the cerebral white matter as a whole. The corpus callosum is uniquely well suited for determining this ratio. EM histology can be used to unambiguously count the total number of callosal axons because the limits of the callosum are well-defined, axons are aligned, and sections can be cut perpendicular to their axis. Likewise, dMRI-derived streamlines can be unambiguously and exhaustively assigned to the callosum because the source and destination of all axons passing through the callosum as a whole are well-defined. With these measures, the axon:streamline ratio is clear for the callosum and the major assumption of our method is that it can be applied to intra-hemispheric fibers. Although related measures are available for intra-hemispheric connections, they are less certain because they lack these features. Consequently we provide several empirical and theoretical results which we believe collectively render it very likely that our main conclusions are substantially correct, including:

1) Histological studies in humans and macaques, and advanced dMRI studies in humans, reported by multiple groups, indicate that callosal and non-callosal fiber tracts have similar axon densities to each other, and furthermore change little both within the callosum and within each hemisphere. Variation in these measures is less than two fold, which would have negligible consequence for our conclusions.
2) Studies in macaques show that dMRI-streamlines and quantitative histological axon-tracing are correlated.
3) Comparison of streamlines vs. distance plots between callosal and non-callosal fibers fail to reveal any of the features that would be predicted if they had differing axon:streamline ratios.
4) Reviewer 1 proposed a specific possibility: that short range (<40mm) axons were more densely packed and less well detected with dMRI. We simulated this possibility and found that it had no effect on our conclusions.
5) We have further quantified the effect of large errors from 50% to 200% of assumed values of critical parameters (packing density and dMRI sensitivity). We simulated errors that were uniform over all distances and those that were proportion to distance. Again, even if these parameters are mis-estimated by up to two-fold the effect on median inter-area axon count is only ~36%, which has no significant effect on our basic conclusions.
6) The volume of hemispheric white matter imposes an absolute constraint on the number/density/length of cortico-cortical axons. Our assumption (supported by existing data) that callosal and non-callosal connections have similar axon-densities and axon-streamline-ratios results in a total volume fitting within that constraint. Changes in any one of these parameters requires that the others change in order to continue to comply. This constraint requires that if there is an error in one of our parameters that there must be balancing errors in other parameters, and thus renders such errors more unlikely. We quantitatively evaluate this constraint and find that even with the unlikely assumption that the relevant parameters are mis-estimated in such a way as to precisely balance each other,
7) We tested the effects of errors in estimating tissue shrinkage and as Reviewer 3 predicted, it did not change our conclusions.

Some of the above considerations were already discussed in our previous submission, but we have developed them and added additional references. We believe that we make a compelling case that there is no reasonable alternative set of assumptions that would lead to a different conclusion regarding how sparse long-distance inter-connections are in the human cortex.

We fully appreciate your caution in publishing a claim that is likely to be widely cited and alter current conceptions of cortical integration. As an example, with your permission, we have added a short analysis estimating the proportion of arcuate and superior longitudinal fasciculus axons that run the entire distance between anterior and posterior language areas. Depending on the assumptions, only ~1 to 5% of the axons in a middle section of these fasciculi directly connect Broca's and Wernicke's areas. It has previously been noted that some white matter tracks may be similar to a trans-continental interstate, with cars entering and leaving for short trips along the way but few traveling from coast-to-coast. We believe that this is the first time that this effect has been quantified for intra-hemispheric tracts.

In addition to the consensus question of extrapolation from the callosum described above, and various lesser critiques, each reviewer had a distinct major concern summarized here briefly. Reviewer 1 expressed concern over the methodology used for dMRI tractography itself, which we have addressed by repeating our analysis using tractography data from Arnatkeviciute et al. (2021) *Nature Communications*, yielding very similar results. Reviewer 2 raised the interpretational concern that we had been reductive in equating effective connectivity with only the quantity of pairwise fibers, discounting the qualitative diversity among axons. Our discussion has been broadened to consider this important distinction. And Reviewer 3 noted that we a misunderstood the magnitude of tissue shrinkage caused by different sample fixation procedures resulting in an approximately two-fold misestimation, which has been duly corrected.

Below, we have responded to reviewers' concerns point by point and hope that the revised manuscript is now considered fit for publication.

Reviewer #1:

The authors present a potentially interesting paper that proposes a relationship between probabilistic tractography streamlines and absolute axon counts after having related the data to the prior histological literature, particularly Aboitiz et al., 1992, but also Arcelli et al., 1997 and Liewald et al., 2014. I am not convinced of the authors' claims at this point.

Major Concerns:

1) The authors cite Donahue et al., 2016 Journal of Neuroscience as evidence of a strong relationship between histological and tractography connection strengths. However, their data shows a strong correlation only for the short distance strong connections (see their Figs 4A and 5). Long-distance (e.g., such as the corpus callosum) and weak connections had many discrepancies between tractography and tracers (see Figure 4a). Thus, I am not sure that this citation is good evidence for the authors' point.

Overall, Donahue et al. [1] found that log-transformed dMRI tractography streamline counts explain ~35% of the variance in log-transformed retrograde tracer data. Clearly there is uncertainty in the relationship, and some measurement error in both datatypes. The reviewer correctly notes that the relationship is less consistent for longer tracts. Although Donahue's report was limited to intrahemispheric connectivity, we find no evidence that distance-matched intra- and inter-hemispheric connections are systematically different (Figure 1C of our manuscript). Figure 5B of Donahue et al. could be interpreted as indicating that dMRI tractography tends to underestimate connectivity weights at longer tract lengths relative to histology, though the trend is not monotonic and unfortunately the confidence intervals of the medians are not shown so it is difficult to interpret how different the distributions of weights within length bins are. However, these data only include connections which were non-null in both the dMRI and histological datasets. Panel A of the same figure shows that the number of dMRI-derived non-null connections which are not found histologically increases with tract distance and, except for a small dip the last length bin, this trend is monotonic. Thus, the data in figure 5A indicates that, relative to histological tracing, dMRI tends to overestimate the presence of long-range connections. This implies that long range cortical connectivity may be even sparser than our dMRI-based evidence indicates, which reinforces our central conclusions.

Furthermore, Donahue and colleagues concluded that "monkey tractography connection weight estimates are accurate to within one or two orders of magnitude relative to tracer-based ground truth suggest that quantitative studies in humans may be limited to an approximately comparable degree using high-quality data such as that from the HCP" . Another comparison of dMRI tractography and histological tracing in macaques, van den Heuvel et al. (2015) [2], concluded that "the number and density of tractography streamlines of DWI reconstructions of macroscale connectome pathways ~~to~~ form a valid *in vivo* approximation of white matter tract strength". As reviewer 3 noted, in the sort of statistical neuroanatomy we present here, our claims are of order-of-magnitude precision. Estimates of such precision are still useful as interareal connectivity varies over more than seven orders of magnitude. We have edited the

text to clarify this point. In addition, we have supplemented the Donahue et al. citation with van den Heuvel et al. reference, and tempered our language concerning the evidence for equivalence of dMRI tractography and histological tracing in macaques.

2) The authors use a single value for the total number of axons crossing the corpus callosum; however, it is known (e.g., shown in Aboitiz et al., 1992) that there are major differences in axonal density in different parts of the corpus callosum, with larger, lower density axons in the posterior midbody and posterior splenium of the corpus callosum with higher axonal density in the genu and rostrum. If we are to believe that tractography streamlines can be scaled to represent axonal density, we would expect a similar pattern to be present in the tractography data as the axons cross the midsagittal plane of the corpus callosum. Was this indeed the case in the study carried out by the authors? Also, it is not clear to me that the authors have internalized this important point from Aboitiz et al., given the statement "However, the major fasciculi (including the corpus callosum) have similar axon calibers and packing densities," as it would seem that the corpus callosum at least is not a good example of such properties.

As reported by Aboitiz et al. [3], the axonal densities vary by a factor of 0.75 at most across the corpus callosum (~3e5 to ~4e5 axons/mm^2 as counted with the Holmes stain, shrinkage uncorrected, Fig. 1B). Our simulations show such variability has very minor effects on the median inter-areal connectivity (~10%) and thus no consequence for our conclusions. As shown in figure S5A doubling or halving the assumption-derived scalar only nudges the distribution slightly.

3) It was not immediately clear to me how the authors derived axonal values from Arcelli et al., 1997 and Liewald et al., 2014 on a quick review of these papers.

Liewald et al. [4] reports axons counts from three regions of the corpus callosum in two brains (Table 1) and indicates that the total area investigated was 1,352 μm^2 (or 1.352e-3 mm^2) per region. We took the mean of the counts from all regions and both brains, mean(389, 431, 250, 376, 465, 451) = 393.67 axons and divided this by the indicated area, 393.67 axons / 1.352e-3 mm^2 = 2.91e5 axons / mm^2. Correcting the density for our assumed 65% linear shrinkage yields the final figure, 2.91e5 *0.65^2 = 1.23e5 axons / mm^2. Note that we have now updated the tissue shrinkage coefficients and derived values throughout the manuscript to reflect differences between Epon and paraffin fixation, as per Reviewer 3's comments.

The reviewer correctly notes that Arcelli et al. [5] is insufficient to derive the number of thalamocortical axons. The thalamic citation was meant to include both Arcelli et al. and Xuereb et al. 1991 [6]. With Xeureb et al. giving the total thalamic cell counts and Arcelli et al. giving the fraction of these which are excitatory thalamocortical cells. Specifically, in table 3, Xuereb reports 18.24e6 neurons in the right thalamus, excluding the reticular nucleus, zona incerta, limitans/suprageniculate, and subthalamus. Multiplying this value by ~62% of cells Arcelli reports as excitatory and by 2 hemispheres yields ~22.6e6 thalamocortical neurons. The count has be clarified in the appendix and citation omission corrected.

4) I had some concerns about the tractography methods: a) It seems that the authors did not use the surface-based tractography available in probtrackx2 despite the fact that it is more accurate than the 3D volume-based approach (e.g., voxel corners do not stick out into deep white matter) and the 360 cortical areas exist natively on cortical surface meshes. b) More to the point, the authors seem not to have understood how to use the data as released by the HCP for such purposes and produced a convoluted and potentially error prone process for getting the 360 cortical areas aligned with the bedpostX data that requires mapping from the 32k fs_LR space to fsaverage ico5 space, then to the FreeSurfer volume space, and then finally to the bedpostX space. Even if they had chosen not to use surface-based tractography, they could simply have mapped the cortical areas directly from the 32k fs_LR surfaces in the ${StudyFolder}/${Subject}/T1w/fsaverage_LR32k folder to the volume space in the ${StudyFolder}/${Subject}/T1w/Diffusion.bedpostX folder using a single command wb_command -label-to-volume-mapping. A simple question on the HCP-Users mailing list could have clarified the correct approach before large scale computations had been undertaken. c) Similarly, it was not clear how the authors prevented axons from crossing CSF spaces bounded by the pial surface or through the ventricles. Thus, it is not clear that the streamlines being studied here were forced to even cross the corpus callosum.

We agree with the reviewer that surface-based tractography in probtrackx2 [7] would have been preferable to the volumetric approach and regret that this approach was not used in our previous study [8]. The method used for translating the HCP-MMP1.0 parcels [9] into FreeSurfer [10] labels comes from the official HCP FAQ [11]. Having FreeSurfer formatted labels also facilitated some auxiliary measurements (e.g. area and adjacency), though it is true that these computations could be performed with workbench formatted files. And while exclusion masks for the ventricles were not explicitly applied, spot inspection of the fdt_paths volumes (averaged across all seed labels) did not show obvious incursions into them. However, it is fair to say that the tractography approach used was generous when assigning streamlines. As discussed in our previous study [8] probabilistic tractography in general and unguided (i.e. without waypoint masks) probtrackx2 in particular is biased towards false positives vs. false negatives. Because in our previous report we were interested in building a data-driven connection matrix of all cortical areas, rather than just the connectivity 'backbone' as is often done, this bias was more acceptable than the reverse. Given the generous nature of the tractography, our central finding of a sparsely connect cortex is all the more remarkable, as a more conservative approach would likely result in even fewer interareal axons.

In order to demonstrate that the key principles and findings of this report are robust to the details of tractography procedure, we repeated our analysis on the tractography data of Arnatkeviciute et al. [12], whose data are available at https://doi.org/10.5281/zenodo.4733297. These data consist of a 972 subject subset of the 1,065 HCP subjects we analyzed and are also organized into the HCP-MMP1.0 360 area parcellation [9]. However, tractography was performed with the MRtrix3 tool [13] as opposed to probtrackX2 [7]. When confined to the same subject subset and non-null connections, the pairwise tract lengths and log-transformed streamline counts are highly correlated ($r^2 = 0.78$ and $r^2 = 0.69$, respectively) between their study and ours. The rate of exponential decay in the number of streamlines vs fiber tract distance is also similar, $\lambda = 26.1$ mm in their study vs 23.4 mm in ours, though less connectivity variance is explained by distance in their results ($r^2 = 0.12$ vs. $r^2 = 0.52$). In our replication analysis with the Arnatkeviciute data (presented in Figure S2) we found median pairwise axons counts were reduced, though by less than an order of magnitude. Thus, the central conclusions of our paper are not dependent on the details of the tractography analysis performed. Indeed, to the degree that the tractography procedure we used in our previous paper [8] was too liberal in assigning streamlines, then the long range connectivity in the cortex is even more sparsely connected than we have calculated in this report.

5) Inadequate consideration of alternative explanations. The authors state key assumptions regarding fiber diameter distributions and the 'conversion ratio' in several places:

a. line 81 "Note that this procedure does not at all depend on the scale of the dMRI metric, requiring only that it be proportional to absolute number of fibers."

b. line 84: "It only requires that the dMRI method return a continuous distribution of connectivity values and have reasonably similar sensitivity to callosal and ipsilateral fiber tracts."

c. Line 153 "While we derive a single dMRI-to-axon count factor, it is possible that the true conversion ratio varies somewhat among connections due to local microstructural differences other than axon count such as axon caliber, packing density, or myelination. However, the major fasciculi (including the corpus callosum) have similar axon calibers and packing densities [12]."

The main experimental support of these statements is the Liewald et al. (2014) EM study reporting roughly similar fiber diameter distributions for two large intrahemispheric tracts compared to the callosum. However, an alternative hypothesis is that short-distance pathways, particularly those connecting sulcal banks with a thin white matter gyral blade in between are mediated by axons having substantially smaller average diameter. Suppose, for example, that there is a two-fold difference in average diameter for axons shorter than ~4 cm (never reaching the major tracts) vs axons that do reach major tracts. Since these account for the majority of intrahemispheric connections (Fig. 1C), this would imply a many-fold greater number of inter-areal axons than the 1.05 x 10^9 value proposed by the authors. In principle, the disparity between short and long distance axonal diameters could be even greater. Hence, without high quality empirical data on axonal diameters as a function of axonal length, it behooves the authors to be far more circumspect in their claims.

First we note that the axon count estimate does not actually require that the packing densities of the corpus callosum and ipsilateral white matter be the same, but the lesser assumption that the axon to dMRI streamline ratio be reasonably constant for intra- vs inter-hemispheric connectivity. It is true, however that if the packing densities of ipsilateral and callosal long distance connections are also similar then the total hemispheric white matter volume provides an absolute constraint on the number of long distance connections that provides a powerful validation of our estimate.

The literature provides converging evidence that ipsilateral and callosal packing densities are indeed similar. In addition to the study of Liewald and colleagues [4], Zikopolous and Barbas [14] found that the average cross-sectional packing density of human prefrontal white matter (immediately sub-gray) is remarkably similar to callosal values (3.5e5 myelinated axons/mm^2, shrinkage corrected). Note that these measures would sample the shorter connections referenced by reviewer 1, as well as longer connections. Furthermore, in a follow-up study they report that this axonal density immediately below the cortical gray-white interface varies among cortical regions by a less than factor of 2 [15], not unlike the density of the callosum itself. While dMRI-based estimates of axon density and caliber are imperfect, they find that axon density varies by less than 2-fold both between major ipsilateral tracts and within each tract along their length [16]. Axon diameter, as estimated with dMRI, likewise varies by 20% among ipsilateral tracts and by at most a factor of 2.2 among white matter voxels, including those of the corpus callosum [17,18]. Likewise, distributions of dMRI-derived axon diameter for the callosum [19] and whole cerebrum [18] are similar with the bulk of values between 2.5 and 5 μm. High quality histological measurements in macaques concur that axon diameters are very similar within the callosal and non-callosal segments of major fasciculi and vary by less than 2-fold across the cortex [20]. This limited variation in diameter is not systematically dependent on the length of axons but rather by their regions of origin and termination [21]. Thus, dMRI-based estimates and direct histological measurements by two groups in humans, as well as histological measures in macaques, all find that callosal and intrahemispheric axonal density are similar.

Furthermore, axonal packing density and the rate of connectivity are co-constrained by the physical volume available for white matter. The white matter volume we calculated based on assuming that axonal packing density, and streamline to axon ratio, are the same for callosal and inter-areal intrahemispheric axons fit quite well within the well-established hemispheric white matter volume, as all acceptable solutions must. Any changes to our estimated values for axonal packing density and streamline-to-axon ratio need to co-vary to arrive at the observed hemispheric white matter volume. Varying each of these assumed values from 50 to 200% of literature values results in minimal changes in our main calculated value of interest, the median inter-parcel axon number.

The reviewer suggests that our dMRI method may have undercounted short fibers. To address this alternative hypothesis directly, we calculated the effect of halving the number of axons represented by each streamline for connections between parcels separated by < 40 mm, or in other words, assumed that dMRI tractography is half as sensitive to these connections. In order to maintain the volume of cortical axons within the measured white matter we also doubledthe axon density. Before doubling the number of short axons, our estimate for the total number of interareal axons is 2.43e9 (after correcting our shrinkage coefficient per reviewer 3's comment). Slightly more than 1% of pairwise interareal connections are <40 mm in length and these connections contain 37.5% of the total estimated interareal axons and therefore doubling them yields 3.3e9 total interareal axons, a 37.5% increase. These areal pairs are universally in the far right tail of the log-normal distribution across parcel-pairs. This relatively minor adjustment to the shortest interareal connections does not affect our central finding that long-range corticocortical connectivity is quite sparse. Specifically relevant to our principal concern, the median inter-areal connectivity does not change whatsoever, because the rank-order of observations is not affected by uniformly increasing the values in the right tail of the distribution. Examining the hypothesis that dMRI tractography is less sensitive to shorter connections more broadly, we increased dMRI streamline-to-axon ratio linearly with inverse fiber length using a range of slope parameters, see figure S5. Packing density was assumed to be reciprocally decreased in order to yield a constant total cerebral white matter volume. Large adjustments resulting in double the axons at the shortest distances only modify median intra-hemispheric axon counts by only ~36% which does not alter our main conclusions.

We certainly agree with the reviewer that additional studies directly comparing dMRI-based streamline counts to histologically quantified axon counts would be very welcome. We recognize that differential dMRI sensitivity could vary between the corpus callosum and hemispheric white matter due to differences in their degree of myelination, volume of glia, interstitial space, etc. as well as the true degree of (an)isotropy of the fibers in any particular cross-sectional plane. However, several lines of evidence, including those detailed above, support our critical assumptions and indicate that even large errors in their estimates would not substantively change our conclusions. We have now included these additional references, calculations, and detailed arguments in the manuscript.

6) Issues of scaling. There is an apparent mismatch between the reported tractography-based streamline distances and the physical size of the human brain.

a. Line 112: "The mean fiber tract lengths of connections were taken from our prior dMRI analysis [3]." This mean length is not stated in the present ms and does not appear to have been mentioned in ref 3. Please state what that value is.

The volume of all pairwise connections are calculated serially by multiplying the inverse packing density, which we treated as a constant, by the fiber tract length of each pairwise connection. Thus, the mean (across subjects) connection lengths are not a single value but consist of symmetric 360 x 360 matrix with 64,620 pairwise elements. The individual subject matrices as well as the average fiber tract length matrix are available at https://doi.org/10.5281/zenodo.4060485 in matlab v7.3 format.

b. Line 145 "Specifically, the corpus callosum is a distinct tract for only about 15-35 mm, but its fibers range in length up to about 300 mm". This is consistent with Fig 1C of the current study, which shows a maximum length >300mm for interhemispheric and >250mm for intrahemispheric fiber tract distance. This is very puzzling insofar as the maximum tract length shown in Fig. 7 of Rosen & Halgren 2020 is <180 mm, which is consistent with the known A-P length of human brains. The authors need to explain the apparent discrepancy between the two studies and to provide evidence that these extreme lengths represent plausible anatomical trajectories within the white matter.

In our data, the longest mean interparcel tract distance is 247.4 mm in the left hemisphere (V1 <-> IFJp), 254.3 mm in the right hemisphere (IFJp <-> VMV1), and 307.1 mm) for interhemispheric connections (right V8 <-> left IFJa). For comparison, the maximum mean intrahemispheric fiber tract distance in Arnatkeviciute et al. [12] is 244.8 mm. Interparcel tract distances with lengths > 200 mm are relatively rare but are clearly visible in Figure 2 (as well as figure 3 and extended data figures 1-1 and 2-1) of Rosen & Halgren [8]. Figure 7 in that paper shows distances from parcels in a Language/Auditory functional network to ipsilateral parcels both within and outside that network. As the Language/Auditory network is fairly centrally located on both A-P and S-I axes of the cortex one would expect the maximum length from these areas to be somewhat shorter than the longest possible intrahemispheric paths. In addition, because Figure 7 is intended to compare distance-matched connectivity within vs. between functional network connections, its domain is bounded by the maximum length of the former and minimum length of the latter. The known A-P lengths you refer to are perhaps Euclidean distances. For our HCP cohort, the mean maximum Euclidean distance between vertices on the left hemisphere reconstructed gray—white interface is 168.1 mm. Fiber tract distances between areas are longer than Euclidean distances due to their curving trajectories. In macaques, the lengths and trajectories of dMRI tractography and histological tracing have been found to have good correspondence [22,23].

7) Confusing wording. Line 145 "Of course, short interhemispheric trajectories are dominated by the callosal segment, and long by the intrahemispheric segments. Generally, the proportion of their trajectories that are within the callosum is roughly proportional to their length. Consequently, if there were enhanced detection of callosal fibers as streamlines by dMRI, the green trace in figure 1C would be expected to be elevated primarily at short fiber lengths, resulting in a noticeably steeper slope for the blue than the green trace, which again is not observed. These data support the applicability of the scaling factor derived from interhemispheric fibers to intrahemispheric fibers."
These statements are confusing - please explain them more clearly.

We agree and have rewritten the passage to be more clear.

8) Tractography false positives. Fig 1D: V1 has one of the highest apparent interhemispheric connectivity values among all parcels. Yet in the macaque few if any V1 neurons project contralaterally (Van Essen et al., J. Neuroscience, 1982). Hence, this is a likely example of artifactually large number of false positive connections revealed by tractography.

While the reviewer is correct that V1 has the most interhemispheric axons of any parcel, it is the largest parcel by far. When normalized by its area, V1 has the 57th and 58th most interhemispheric connectivity and 161st and 162st most total axons/mm^2 in the left and right hemispheres, respectively. To address the larger issue of false positive streamlines, please see the discussion above of our replication analysis with the Arnatkeviciute et al. [12] data. Again, any possible systematic overestimate of connectivity with tractography further emphasizes the sparsity of long range connections in the cortex.

Reviewer # 2, Basilis Zikopoulos:

The paper entitled "Human cortical areas are sparsely connected: Combining histology with diffusion MRI to estimate the absolute number of axons" provides estimates of the number of long-range axons connecting cortical areas within and between hemispheres, using the myelinated fiber density of the corpus callosum, measured histologically, to calibrate whole-cortex diffusion MRI (dMRI) connectivity data. The authors conclude that cortical areas within hemispheres are sparsely connected, with an average of about 2,700 axons, whereas connections of areas between hemispheres involve about five times fewer axons.

This is a very timely, interesting, straight-forward, and well written study and I want to commend the authors on their excellent idea to perform this, much needed work, and analysis. The strengths of this manuscript include (1) the high quality of the data analyzed (from detailed high-resolution histological studies and a large dMRI dataset) and (2) the theoretical framework used to correlate data from sets at multiple scales for the analysis, which is based on the key relationship between structural features and connectivity of the cortex.

The main apparent limitations that will need to be addressed in this or future experimental and theoretical work include the lack of cross-validation of the findings using additional approaches for calibration and combination of the two datasets, the dearth of high-quality, high-resolution histological data, and the low resolution and threshold sensitivity, especially of the dMRI dataset for the detection of short- and some medium-range connections that also include significant numbers of thin, branching, and unmyelinated axons. Despite these limitations, the findings are novel, highly significant, and the manuscript is poised to be an outstanding contribution to our field and of general interest.

Below few comments and suggestions that in my opinion will further increase the value and clarity of the manuscript, strengthen reported findings, and place them in the context of key principles that underlie functional and structural cortical network organization and connectivity:

1. The last sentence in the abstract (lines 59-61) and relevant discussion part (last paragraph, lines 180-188) are somewhat problematic, or a narrow interpretation that can be misinterpreted or misleading and should be modified. The authors' interpretation that the sparseness of long-range connectivity suggests that cortical integration relies mainly on extremely dense local connections and that models that require direct long-range connectivity are somehow challenged by these findings, is not justified, because that would assume that all pathway interactions with different types of excitatory and inhibitory neurons, receptors, distal or proximal dendritic segments, numbers of axon branches and terminals are similar across the cortex. Another equally plausible interpretation based on these findings would be that sparse, in terms of axons, long-range connectivity can still produce major effects postsynaptically, which can integrate information and direct cortical activity, due to specialized interactions with key elements of local circuits. In addition, the last statement of the abstract also suggests that direct transmission of information between cortical areas may be substituted by indirect connections (serial multisynaptic steps of short-range connections) overlooking differences in conduction velocity and variable key interactions with

distinct local inhibitory and other microenvironments for each set of pathways. As such, the following statement in the discussion "In such models, cortical locations interact through activation of multi-synaptic pathways rather than direct connections, and the key to connectivity is physiological selection under multiple constraints rather than anatomical projections" wrongly implies that long-range pathways do not lead to connectivity that is physiologically selected under multiple constraints and complex interactions, and should be modified.

We concur with the reviewer that we discounted the possibility that long-range connections, despite being few in number, may have a disproportionate impact on cortical processing [24]. Though the two are undoubtably related, we were overly reductive in equating the number of pairwise axons with the degree of physiological coordination between areas. As the reviewer correctly notes, axons may have various morphological or molecular properties which amplify or diminish their influence on information processing relative to each other, and thus the possibility of outsized effects of the rare long-range axons cannot be discounted. We have updated the text in abstract and discussion to reflect these considerations.

2. Methods and Results sections: the authors compare linear regressions of distance-matched streamlines to find no difference between inter- and intrahemispheric connections, as shown in Fig. 1C. Even though this is a previously used approach, it must be noted that although distance is often correlated with the strength and/or presence of connections in some studies, it is well-established that it doesn't accurately and fully capture the relationship between connections, and in many cases, it falls apart when describing long-range connections, especially between frontal and parietal lobes, which together constitute a large chunk of the cortex. On the other hand, structural (dis)similarity between areas is a much better predictor of connectivity strength in most mammals studied, including primates, as shown in several studies using golden-standard tract-tracing and structural imaging approaches (see relevant work by Hilgetag, Barbas, Zikopoulos etc.). As the authors likely know, the Structural Model for Connections, also known as Architectonic Type Principle, was initially proposed by Helen Barbas after multiple tract tracing studies in non-human primates; later, this model was extended to other mammalian species and, since then, predictions based on the Structural Model have been consistently confirmed across all cortical lobes and systems in non-human primates and other species. Therefore, we can assume that the relational principle of the Structural Model obtained from animal research applies to the human cerebral cortex. Based on this large body of work we know that cortical areas tend to be connected primarily with other areas that are relatively similar in type (structure etc.), most of which happen to be nearby, but some are quite distant, as for example in the case of the relatively strong connections between lateral prefrontal and parietal cortices that do not fit and overrun distance models. Therefore, several tract tracing studies in non-human primates show that long-range cortico-cortical connections across lobes are far from weak and do involve lots of axons. See for instance the summary figures (Figures 15 & 16) in Cavada & Goldman-Rakic, 1989 J Comp Neurol 287: 422-445. Cavada and Goldman-Rakic showed that projections from prefrontal areas to posterior parietal areas, which are long range connections, are denser than short-range projections from other areas that are closer to the parietal areas injected by these authors. Actually, dense long-range cortico-cortical connections have been shown for multiple areas across the cerebral cortex of the macaque (see Morecraft et al 2004 J Comp Neurol for posterior cingulate and posterior parietal areas, Morecraft et al 2012 Brain Res Bull for frontal motor and anterior cingulate areas, Morecraft et al 2015 Brain Res Bull for insular and parietal somatosensory areas; Zikopoulos et al 2018 PLOS Biol for prefrontal connections with all other lobes; Cavada et al 2000 Cerebral Cortex for orbital areas; Medalla and Barbas, 2006 for frontal and parietal areas; or Joyce and Barbas, 2018 for especially strong long-range connectivity between anterior cingulate areas in the frontal lobe and area prostriata in the occipital lobe of primates). Based on connectivity patterns in primates, the laminar architecture of the cortex, and the principles of the Structural Model, Zikopoulos et al. in 2018 (PLOS Biol) showed that eulaminate areas have comparatively more and stronger long-range connections than limbic cortices and went one step further to predict that this would also apply to the human cortex, especially since the human cortex includes more eulaminate cortices. These findings from tract tracing studies should be taken into consideration and since this study deals primarily with long-range connectivity it would be appropriate and more accurate to correlate dMRI connectivity with structural features of the connected parcels, if possible. Several recent studies in humans have parcellated the human cortex using relevant structural features that could be used to

explore dMRI connectivity relationships. If not possible, at the very least, this should be briefly noted in the Discussion or Appendix.

First, we would like to thank the reviewer for providing these important references. We are aware and have great respect for the structural model of primate brain connectivity [25,26]. In our prior report presenting the HCP dMRI connectome [8] we sought evidence for the model in two ways. First we compared distance-matched connectivity within the language/auditory network and between this network and the rest of the cortex. We found a marked, left-lateralized increase in connectivity within the language/auditory network in ~100-140 mm connection, corresponding to connections between frontal and temporoparietal language areas. Next, we used pair-wise differences in non-invasively determined bulk myelination [27] as a proxy for architectonic similarity to investigate the influence of structural, or hierarchical, similarity on connectivity, figure 8 [8]. We found that overall, the degree of structural similarity of areas weakly but consistently correlates to strength of dMRI connectivity between them. This relationship is considerably stronger when examined within predominantly frontal, functionally related networks such as the right dorsal attention, left language, and bilateral frontoparietal network. However, when quantitatively comparing the degree to which fiber tract distance and architectonic similarly affect connectivity, the balance of evidence in our data favors a model where the former dominates and the latter modulates, as has also been proposed based on histological tracing in macaques [28] (which is of course the same data, from [29] , as [25]). It may be that the non-invasive myelination index we used insufficiently captures laminar similarity. But even Zikopolous et al. (2018) shows that distance and structural similarity have similar degrees of influence (Fig. 11C,D). In any case, the conclusions in our previous report [8] with regard to the influences of distance and architectonic similarity on connectivity are essentially unchanged by the present manuscript as these pertain to relative rather than absolute connectivity. We have added a passage to the final paragraph of the discussion noting the presence of uncommon long-range connections, especially between architectonically similar areas, and that they may have an influence on corticocortical communication disproportionate to their number [24].

3. The axon density in the corpus callosum estimated in previous histological studies (Aboitiz et al., or Liewald et al.) was used and was combined with dMRI data to estimate axon numbers in pathways. The second study referenced has also reported axon density in other major long-distance pathways of the brain, including the superior longitudinal fasciculus or the uncinate fasciculus. In addition, several other high-resolution histopathology studies at the light and electron microscopic level have examined and reported axon features and density in the white matter below prefrontal and temporal cortices (e.g. Zikopoulos and colleagues in 2010, or 2018; Liu and Schumann, 2014) that participate in short- or long-range cortical connections. Combined, some of these data on other major white matter pathways in the human brain could be used to calibrate dMRI connectivity data and cross-validate estimates derived from the callosal calibration. Some of these studies also include very relevant information on the relative prevalence of short- vs long-range connections that could support and strengthen the authors' findings.

The reviewers' request to validate our procedure is well-founded, but it is quite difficult to repeat our calibration analysis with the ipsilateral fasciculi. The corpus callosum is unique in that it has a well-defined cross-sectional area, and more than ~99% of interhemispheric corticocortical axons are routed through it [3,30]. Critically, no fibers enter or exit off from the callosum between the two hemispheres. Therefore, all of the dMRI streamlines between the areas of the left and right hemispheres can be said to travel though the corpus callosum, and nothing else. This discounts the other commissures and the possibility of spurious streamlines across the longitudinal fissure, but these are very minor contributors. A similar mapping cannot be made of other fasciculi as any particular region of cortex receives (or projects) axons from multiple tracts. For example, in the arcuate and superior longitudinal fasciculi (AF/SLF) most axons exit (or enter) along the course of the tract, i.e. the central tracts are more like highways with many exits and entrances between their origin and termination, than tunnels where all traffic is trans-terminal. In a supplementary analysis using data from the literature as well as our interareal axon counts, we estimated the that only ~1-5% of AF/SLF axons are trans-terminal (see S1 appendix and figure S4).

Furthermore, our count of axons does not rely on the ipsilateral and commissural tracts having similar axon packing densities. It instead only requires that the sensitivity of dMRI tractography to axons contained in these tracts be reasonably similar. We provided evidence for this assumption by comparing

distance matched streamline counts between intra- and interhemispheric connections (Figures 1C and S2C). As we have clarified in the discussion, if a substantive discrepancy existed then it would result in a notable shift in the slope or intercept in the distance vs. streamline traces and this is not observed.

The secondary estimate of the total volume occupied by the interareal axons does assume that the axon packing densities are roughly the same and there is some evidence for this. In addition to the study of Liewald and colleagues [4], Zikopolous and Barbas [14] found that the average cross-sectional packing density of human prefrontal white matter (immediately sub-gray) is remarkably similar to callosal values (3.5e5 myelinated axons/mm^2, shrinkage corrected) and in a follow-up study reports that this varies among the prefrontal regions by a less than factor of 2 [15]. In addition, while dMRI-based estimates of axon density and caliber are imperfect, it has been shown axon density varies by less than 2-fold both between major ipsilateral tracts and within each tract along their length [16]. Axon diameter, as estimated with dMRI, likewise varies by 20% among ipsilateral tracts and by at most a factor of 2.2 among white matter voxels, including those of the corpus callosum [17,18]. And distributions of dMRI-derived axon diameter for the callosum [19] and whole cerebrum [18] are similar with the bulk of values between 2.5 and 5 μm. High quality histological measurements in macaques concur that axon diameters are very similar within the callosal and non-callosal segments of major fasciculi and vary by less than 2-fold across the cortex [20]. And this limited variation in diameter is not systematically dependent of the length of axons but rather by their regions of origin and termination [21]. Again, relatively unbiased ~2-fold mis-estimations in the axon counts of various connections due to differential dMRI sensitivity to white matter microstructure would not substantively change our conclusions. It must also be noted that while there is likely a relationship between axon diameter and packing density, the effective packing density (# axons per mm^2 of white matter cross-section) is also influenced by the degree of myelination, volume of glia, interstitial space, etc. as well as the true degree of (an)isotropy of the fibers in any particular cross-sectional plane. We have added this information the discussion. As some regions have somewhat lesser density and others somewhat greater packing density than the corpus callosum, it is likely that errors in the volumetric calculations due to varying packing density are reasonably small and may even mostly cancel when the whole cortex is considered.

4. Discussion, page 7, lines 174-178: this statement can be misconstrued and should be reworded. I recommend stating that <5% of cortical pyramidal cells project outside their immediate neighborhood instead of using the word "area". This is because many short-range white matter connections that are below the resolution of dMRI approaches and are not included in this analysis are between adjacent, relatively small areas or neighboring columns within an area. In addition, a study by Zikopoulos et al., 2018 in PLOS Biology has also shown a clear relationship between the number of neurons and the density of white matter pathways in non-human primates and humans and could be relevant to this statement.

We have replaced the word “area” in the line with “parcel” to make clear that we are referring to the 360 HCP-MMP1.0 [9] areas of the connectivity matrix and added a clause to the end of the sentence specifying that the unaccounted for axons belong to either horizontal or U-fibers and thank the reviewer again for the helpful reference.

5. The authors show that interhemispheric connectivity constitutes on average about 20% of intrahemispheric connectivity (540 vs 2,700 axons). This estimate is in line with similar estimates from Barbas et al., 2005 for non-human primates (estimated that less than 30% of connections are contralateral in rhesus monkeys).

Thank you for pointing out this the intra/inter comparison to us. The Donahue et al. [1] report we cited for validation in macaques unfortunately only investigated ipsilateral connections. We have added your observation and the Barbas et al. citation [31] to the results.

6. Since this study focuses on analysis of long-range connectivity, and uses long-range callosal connections to calibrate dMRI data, it is important to highlight key, relevant differences between long- and short-range connections and U-fibers, something that is now missing. The authors do briefly state that

dMRI methods cannot reliably resolve short-range connections, but I think the readers would appreciate a specific comment in the discussion/supplement regarding key features of pathways, other than axon density and number such as: proportion of thin vs thick axons that correlate well with short- and long-range pathways, proportion of myelinated vs unmyelinated axons (more unmyelinated axons in limbic cortical pathways), myelin thickness data for short- vs long-range connections, which is relevant for conduction velocity and function, axonal branching patterns and size of termination fields that may disproportionately amplify the effects of some connections over others, and how some of these features change as we move from the superficial to the deep white matter (e.g. see Liewald et al., 2014; Zikopoulos and Barbas 2010; Zikopoulos et al., 2018; Caminiti and Innocenti, 2009; LaMantia and Rakic 1990; Makris et al., 1999; Rademacher et al., 1992).

The reviewer correctly notes that the scope of this study is mostly limited to long-range interareal connections, with short-range axons of the U-fiber and horizontal systems only briefly mentioned as subsuming the fibers unaccounted for in our estimate. We also agree that due to their differing properties the callosal calibration technique we present here may require significant modification to be applied to these fibers, even if advances in dMRI allow them to be detected. We have added a remark to this effect to the end of the fifth paragraph of the discussion. In addition, we agree with the reviewer that axons and their post-synaptic influence are not uniform and that the number of axons connecting regions (even if known with certainty) is only a limited proxy for true interareal connectivity. As the reviewer suggests, some axons may exert disproportionate influence by virtue of their morphology (e.g. larger termination fields, greater axonal arborization, or more numerous *en passant* varicosities) or by synaptic specializations. We have now included this caveat in the last paragraph of the discussion.

7. Reference list (page 10): references 5 and 11 refer to the same study and one of them should be deleted, and in-text citations corrected appropriately. Same for references 12 and 14.

The duplication errors in our reference list have been corrected.

8. S1 Appendix, page 14, lines 359-360: The authors state that they used estimates on thalamocortical connections from histological counts in reference [12] - Arcelli P, Frassoni C, Regondi MC, De Biasi S, Spreafico R. GABAergic neurons in mammalian thalamus: a marker of thalamic complexity? Brain Res Bull. 1997;42: 27-37. As I have carefully read this paper several times I am not sure how the authors got estimates about volume of thalamocortical axons and total axon count (22.6x106) from this study, which only reports local inhibition in the thalamus. Perhaps they meant to cite reference [11] instead - Ji JL, Spronk M, Kulkarni K, Repovš G, Anticevic A, Cole MW. Mapping the human brain's cortical subcortical functional network organization. Neuroimage. 2019;185: 35-57. doi:10.1016/j.neuroimage.2018.10.006. Please clarify and correct, as needed.

The reviewer correctly notes that Arcelli et al. [5] is insufficient to derive the number of thalamocortical axons. The thalamic citation was meant to include both Arcelli et al. and Xuereb et al. 1991 [6]. With Xeureb et al. giving the total thalamic cell counts and Arcelli et al. giving the fraction of these which are excitatory thalamocortical cells. Specifically, in table 3, Xuereb reports 18.24e6 neurons in the right thalamus, excluding the reticular nucleus, zona incerta, limitans/suprageniculate, and subthalamus. Multiplying this value by ~62% of cells Arcelli reports as excitatory and by 2 hemispheres yields ~22.6e6 thalamocortical neurons. The count has be clarified in the appendix and citation omission corrected.

Reviewer #3: Almut Schüz,

This is a fascinating paper. It quantifies in an elegant way cortico-cortical connections between distant cortical areas in the human brain. The results are in support of findings which indicate a preponderance of connectivity between closely located areas (Schüz and Braitenberg, 2002), also in other species (Scannell et al., 1995; Schüz at al., 2006). The study by Rosen and Halgren is outstanding since - in contrast to previous studies – it is able to provide an astonishingly concrete estimate for the median number of axons between distant cortical areas in the human brain.

The study uses the parcellation into 180 areas in each hemisphere by Glasser et al. (2016). This parcellation is based on a combination of neuroanatomical (mainly myelin) and functional features, by way of MRI and fMRI. The number of areas comes close to that of the myeloarchitectonic areas by the Vogt and Vogt school. The present study is based on diffusion MRI data from the database of the Human Connectome Project.

In this paper, the relative connectivity provided by dMRI (number of streamlines) is transformed into absolute numbers of axons. This transformation is based on a comparison with histological data from the literature on the density of axons in the Corpus callosum (Aboitiz et al, 1992). It leads to a conversion factor of 0.87 axons per streamline. The authors assume that the same factor can be applied to both, the Corpus callosum and to the other long range systems via the white matter. This is a reasonable assumption.

Presentation of data:

It would be good to visualize not only the median:

The HCP data contain a family structure with genetic related and unrelatedness and many other behavioral measures (Van Essen et al., 2012). The data also vary with age (22-36 years). Thus, the number of streamlines (Page 11, Line 292) between cortical areas shows inter- individual variability, affecting axon estimation. It would be essential to visualize a scatterplot (e.g. for the Corpus callosum) how the spread of the number of axons is depicted in the healthy HCP sample. Are these values in an acceptable range? As tractography relies on coarser spatial resolution, partial volume effects, and may be erroneous due to false- positive/negative estimation of streamlines.

In their histological study of 20 cortices , Aboitiz and colleagues [3] found no significant effects of sex or age on the total number of fibers, fiber density, and cross-sectional area of the corpus callosum. As we assume a constant fiber density, our estimate the total number of interhemispheric fibers is a linear multiple of the callosal cross-sectional area. As the ages of the HCP 1200 cohort are grouped into four broad ranges in the open access data, we investigated the effects of sex and age on the estimated number interhemispheric axons with 2x4 fixed effects ANOVA, treating the age group factor as categorical. Mean and individual values are now shown in figure S3. Note that while the number of pairwise axons is approximately log-normally distributed across areal pairs, it is approximately normally distributed across individuals. As examined with a fixed-effects ANOVA, the effect of sex does not reach significance and the effect of age is driven by the modest difference in total interhemispheric axons between the 22-25 and 26-30 age groups. These results are broadly consistent with those of Aboitiz et al. [3]. A more in-depth examination of the effects of age on corpus callosum requires a cohort which includes more older subjects as well as more precise age values. We had added these methods and results to the appendix S1. In addition, the inter-individual variability of the pair-wise interareal connections was examined in our prior report [8] (fig. 3) and those results are essentially unaffected by the linear unit conversion from relative connectivity to number of axons.

The conversion factor

The conversion factor is the crucial point in this paper. Re-reading Aboitiz' paper and based on my own histological experience I come to the conclusion that your conversion factor is at the lower end and is rather around 1.6. This does not invalidate the paper – a factor of 2 is negligible in this kind of statistical neuroanatomy – but it gives an idea of the possible range.

Let me explain. In Line 307 to 313 you describe your approach. In line 308 you say “electron microscopic study”, but it is both light and electron microscopic. The shrinkage factor mentioned in the method's part of Aboitiz' paper is only valid for his light microscopic material, embedded in paraffin. He does not mention any shrinkage factor for his electron microscopic material (embedded in Epon), and – according to our own experience – there is hardly any shrinkage in such material. (The volume in our EM-material is about 96% of the original tissue after fixation; Schüz and Palm. 1989).

The number you mention for light microscopy of $1.57x10^5/mm^2$ is not mentioned explicitly in Aboitiz paper as far as I can see, but you probably calculated it from the data given in his table I and corrected it for areal shrinkage. Correct?

Aboitiz estimates that about 20% of fibers were not detected in the light microscope. So we end up with a range of about $1.6x10^5/mm^2$ from light microscopy and about $3.8 \times 10^5/mm^2$ from electron microscopy. The reality is probably somewhere between these values.

This is supported when looking at the total number of axons in the Corpus callosum. Aboitiz estimates 2 $x10^8$ fibers. This is twice the number you get when using his light microscopic density of about $1.6x10^5$ mm^2 and your average areal size. (He does not give an areal size as far as I can see). This speaks in favour of a density between the LM and EM-data, and it leads to a conversion factor of 1.6 rather than 0.87.

The inverse packing density (area per axon) in line 104 would then be lower, but well within the possible range. The average axonal diameter is below 1 m in most cortico-cortical long- range systems (Liewald et al, 2014).

Thank you very much for pointing out the differences in tissue shrinkage between tissue preparation for light and electron microscopy, and for the reference. If we substitute the 0.65^2 shrinkage coefficient with $0.96^{2/3}$ for EM data, we get 3.7e5 axons/mm^2 for the Aboitiz et al. [3] EM-derived value and 2.85e5 axons/mm^2 for the (non-exhaustive) Liewald et al. [4] derived figure. Aboitiz and colleagues only corrected for tissue shrinkage when reporting fiber diameters and are explicit when reporting corrected values. The fiber densities they report in table 1 are therefore uncorrected. We arrived at the 1.57e5 axon/mm^2 value by taking the mean Holmes stain derived density of 3.717e5 axons/mm^2 and multiplying by 0.65^2 to correct for shrinkage, though we did not adjust for the ~20% of fibers which are not detected under light microscopy. Including this and using the Schüz & Palm [32] paraffin shrinkage coefficient, the figure becomes $3.717e5 * 0.43^{2/3} / 0.8 = 2.65e5$ axons/mm^2.

Applying the Schüz & Palm shrinkage coefficient yields a axons per streamline conversion factor of 2.0. Again, we note that the quantitative value of the factor is dependent on the dMRI tractography methods and parameters used for dMRI connectome. For example, when we repeat the analysis with the dMRI tractography data from Arnatkeviciute et al. [12], we find the factor to be 137.6, but the derived axons counts are similar. We have cited Schüz & Palm [32] for EM shrinkage and updated all of the calculations and results to reflect these shrinkage values. As the reviewer notes, a factor of ~2 does not affect the overall tenor of our results.

As an aside, Aboitiz gives post-fixation average areas for 10 regions of the callosum in the legend of Fig 1. Summing them yields 429.2 mm^2 and dividing by 0.65^2 to account for shrinkage yields 1,015.9 mm^2 This is 3.3 standard deviations above our mean MRI-derived callosal area 689.4 mm^2. Interestingly, dividing by 0.65^1 yields a more reasonable 660.3 mm^2. While the way Aboitiz corrected fiber diameters for shrinkage implies that his 0.65 factor was linear, this indicates it to be in fact areal. If we set aside the differences of sample, taking the quotient between our HCP MRI-derived (and shrinkage-free) mean callosal area and Aboitiz's mean area, 429.2/689.4 yields an areal coefficient of 0.62, or linear coefficient of 0.79 and volumetric coefficient of 0.49, which are values more consistent with those found in Schüz & Palm.

Some points to be clarified

In the discussion in lines 130 and in line 165 the authors quote Liewald et al. (2014) for an alternative value for packing density in the corpus callosum of 1.23×10^5 /mm2. I cannot find this number in the quoted paper. Did the authors somehow calculate this value from the fiber diameters given there? Or did I overlook something?

Liewald et al. [4]. reports axons counts from three regions of the corpus callosum in two brains (the rightmost column of Tbl. 1) and indicates that the total area investigated was 1,352 μm^2 (or 1.352e-3 mm^2) per region. We took the mean of the counts from all regions and both brains, mean(389, 431, 250, 376, 465, 451) = 393.67 axons and divided this by the indicated area, 393.67 axons / 1.352e-3 mm^2 = 2.91e5 axons/mm^2. Correcting the density for our assumed 65% linear shrinkage yields the final figure, 2.91e5 * 0.65^2 = 1.23e5 axons/mm^2. We have updated this to 2.83 axons/mm^2 using the Schüz & Palm coefficient (2.91e5 * $0.96^{2/3}$). As the paper's primary focus was on axon diameters rather than packing density, we were not surprised that density figure is marginally lower than that of Aboitiz et al. We presume that obliquely transected axons with difficult to measure diameters were not included in the study's count and labeled the alternative packing density "non-exhaustively counted" in our text. Nevertheless, we consider these derived densities a confirmation that the Aboitiz et al. reported callosal density is reasonable.

Another point: in line 176 the authors quote Azevedo et al. (2009) for a number of $11.5x10^9$ cortical pyramidal cells. I cannot find a number for cortical pyramidal cells in this paper. Did the authors derive this from the total number of cortical neurons mentioned on p.535 ($16.34x10^9$) and perhaps subtract a percentage of non-pyramidal cells?

The reviewer is correct, we took the 16.36e9 total number of cortical neurons from Azevedo et al. [33] and multiplied it by the ~70% of neuron that are pyramidal, rounding up to the nearest half-billion. This is on the low end of the range 70-85% range in DeFelipe & Fariñas (1992) [34]. Assuming a greater proportion of pyramidal neurons or less generous rounding yields an even smaller percentage of cells that project outside their area, further re-enforcing our central conclusions. We have added the DeFelipe & Fariñas (1992) citation.

Also, in some cases the same paper is quoted under 2 different numbers in the reference list: Liewald et al. under 12 and 14, Aboitiz et al under 5 and 11.

The duplication errors in our reference list have been corrected.

Finally, on line 107 the names Schüz and Braitenberg are misprinted. (And thanks to this quotation I discovered a serious printing error in our own paper: on p.381, first line, it should be $6x10^9$ not $6x10^3$)

This embarrassing orthographic error has been corrected, apologies.

References

1. Donahue CJ, Sotiropoulos SN, Jbabdi S, Hernandez-Fernandez M, Behrens TE, Dyrby TB, et al. Using Diffusion Tractography to Predict Cortical Connection Strength and Distance: A Quantitative Comparison with Tracers in the Monkey. J Neurosci. 2016;36: 6758–6770. doi:10.1523/JNEUROSCI.0493-16.2016
2. van den Heuvel MP, de Reus MA, Feldman Barrett L, Scholtens LH, Coopmans FMT, Schmidt R, et al. Comparison of diffusion tractography and tract-tracing measures of connectivity strength in rhesus macaque connectome. Hum Brain Mapp. 2015;36: 3064–3075. doi:10.1002/hbm.22828
3. Aboitiz F, Scheibel AB, Fisher RS, Zaidel E. Fiber composition of the human corpus callosum. Brain Res. 1992;598: 143–153.
4. Liewald D, Miller R, Logothetis N, Wagner H-J, Schüz A. Distribution of axon diameters in cortical white matter: an electron-microscopic study on three human brains and a macaque. Biol Cybern. 2014;108: 541–557.
5. Arcelli P, Frassoni C, Regondi MC, De Biasi S, Spreafico R. GABAergic neurons in mammalian thalamus: a marker of thalamic complexity? Brain Res Bull. 1997;42: 27–37.
6. Xuereb JH, Perry RH, Candy JM, Perry EK, Bonham JR. Nerve cell Loss in the thalamus in Alzheimer's disease and Parkinson's disease. Brain. 1991;114: 1363–1379.
7. Behrens TEJ, Berg HJ, Jbabdi S, Rushworth MFS, Woolrich MW. Probabilistic diffusion tractography with multiple fibre orientations: What can we gain? Neuroimage. 2007;34: 144–155. doi:10.1016/j.neuroimage.2006.09.018
8. Rosen BQ, Halgren E. A Whole-Cortex Probabilistic Diffusion Tractography Connectome. eNeuro. 2021;8: ENEURO.0416-20.2020. doi:10.1523/ENEURO.0416-20.2020
9. Glasser MF, Coalson TS, Robinson EC, Hacker CD, Harwell J, Yacoub E, et al. A multi-modal parcellation of human cerebral cortex. Nature. 2016. doi:10.1038/nature18933

10. Fischl B. FreeSurfer. Neuroimage. 2012;62: 774–781.
11. Coalson T, Van Essen D, Glasser M. hcp-users FAQ #9: How do I map data between FreeSurfer and HCP? 2016. Available: https://wiki.humanconnectome.org/download/attachments/63078513/Resampling-FreeSurfer-HCP.pdf?version=1&modificationDate=1472225460934&api=v2
12. Arnatkeviciute A, Fulcher BD, Oldham S, Tiego J, Paquola C, Gerring Z, et al. Genetic influences on hub connectivity of the human connectome. Nat Commun. 2021;12: 4237. doi:10.1038/s41467-021-24306-2
13. Tournier JD, Smith R, Raffelt D, Tabbara R, Dhollander T, Pietsch M, et al. MRtrix3: A fast, flexible and open software framework for medical image processing and visualisation. Neuroimage. 2019;202: 116137. doi:10.1016/j.neuroimage.2019.116137
14. Zikopoulos B, Barbas H. Changes in prefrontal axons may disrupt the network in autism. J Neurosci. 2010;30: 14595–14609.
15. Zikopoulos B, García-Cabezas MÁ, Barbas H. Parallel trends in cortical gray and white matter architecture and connections in primates allow fine study of pathways in humans and reveal network disruptions in autism. PLoS Biol. 2018;16: e2004559.
16. De Santis S, Drakesmith M, Bells S, Assaf Y, Jones DK. Why diffusion tensor MRI does well only some of the time: variance and covariance of white matter tissue microstructure attributes in the living human brain. Neuroimage. 2014;89: 35–44.
17. Huang SY, Tian Q, Fan Q, Witzel T, Wichtmann B, McNab JA, et al. High-gradient diffusion MRI reveals distinct estimates of axon diameter index within different white matter tracts in the in vivo human brain. Brain Struct Funct. 2020;225: 1277–1291. doi:10.1007/s00429-019-01961-2
18. Fan Q, Nummenmaa A, Witzel T, Ohringer N, Tian Q, Setsompop K, et al. Axon diameter index estimation independent of fiber orientation distribution using high-gradient diffusion MRI. Neuroimage. 2020;222: 117197.
19. Horowitz A, Barazany D, Tavor I, Bernstein M, Yovel G, Assaf Y. In vivo correlation between axon diameter and conduction velocity in the human brain. Brain Struct Funct. 2015;220: 1777–1788.
20. Tomasi S, Caminiti R, Innocenti GM. Areal differences in diameter and length of corticofugal projections. Cereb Cortex. 2012;22: 1463–1472.
21. Innocenti GM, Vercelli A, Caminiti R. The Diameter of Cortical Axons Depends Both on the Area of Origin and Target. Cereb Cortex. 2014;24: 2178–2188. doi:10.1093/cercor/bht070
22. Caminiti R, Carducci F, Piervincenzi C, Battaglia-Mayer A, Confalone G, Visco-Comandini F, et al. Diameter, length, speed, and conduction delay of callosal axons in macaque monkeys and humans: comparing data from histology and magnetic resonance imaging diffusion tractography. J Neurosci. 2013;33: 14501–14511.
23. Jbabdi S, Lehman JF, Haber SN, Behrens TE. Human and Monkey Ventral Prefrontal Fibers Use the Same Organizational Principles to Reach Their Targets: Tracing versus Tractography. J Neurosci. 2013;33: 3190 LP – 3201. doi:10.1523/JNEUROSCI.2457-12.2013
24. Deco G, Sanz Perl Y, Vuust P, Tagliazucchi E, Kennedy H, Kringelbach ML. Rare long-range cortical connections enhance human information processing. Curr Biol. 2021;31: 4436-4448.e5. doi:https://doi.org/10.1016/j.cub.2021.07.064
25. Beul SF, Barbas H, Hilgetag CC. A Predictive Structural Model of the Primate Connectome. Sci Rep. 2017;7: 1–12. doi:10.1038/srep43176
26. Barbas H. General Cortical and Special Prefrontal Connections: Principles from Structure to Function. Annu Rev Neurosci. 2015;38: 269–289. doi:10.1146/annurev-neuro-071714-033936
27. Glasser MF, Van Essen DC. Mapping Human Cortical Areas In Vivo Based on Myelin Content as Revealed by T1- and T2-Weighted MRI. J Neurosci. 2011;31: 11597–11616. doi:10.1523/JNEUROSCI.2180-11.2011
28. Markov NT, Ercsey-Ravasz M, Van Essen DC, Knoblauch K, Toroczkai Z, Kennedy H. Cortical high-density counterstream architectures. Science. 2013. pp. 1238406–1238406. doi:10.1126/science.1238406
29. Markov, Ercsey-Ravasz MM, Ribeiro Gomes AR, Lamy C, Magrou L, Vezoli J, et al. A weighted and directed interareal connectivity matrix for macaque cerebral cortex. Cereb cortex. 2014;24: 17–36.
30. Highley JR, Esiri MM, McDonald B, Roberts HC, Walker MA, Crow TJ. The size and fiber composition of the anterior commissure with respect to gender and schizophrenia. Biol Psychiatry. 1999;45: 1120–1127.
31. Barbas H, Hilgetag CC, Saha S, Dermon CR, Suski JL. Parallel organization of contralateral and ipsilateral prefrontal cortical projections in the rhesus monkey. BMC Neurosci. 2005;6: 32. doi:10.1186/1471-2202-6-32
32. Schüz A, Palm G. Density of neurons and synapses in the cerebral cortex of the mouse. J Comp Neurol. 1989;286: 442–455.
33. Azevedo FAC, Carvalho LRB, Grinberg LT, Farfel JM, Ferretti REL, Leite REP, et al. Equal numbers of neuronal and nonneuronal cells make the human brain an isometrically scaled-up primate brain. J Comp Neurol. 2009;513: 532–541. doi:10.1002/cne.21974
34. DeFelipe J, Fariñas I. The pyramidal neuron of the cerebral cortex: Morphological and chemical characteristics of the synaptic inputs. Prog Neurobiol. 1992;39: 563–607. doi:10.1016/0301-0082(92)90015-7
